# Socioeconomic inequality and access to emergency care: understanding the pathways to the emergency department in the UK

**DOI:** 10.1136/bmjopen-2025-108770

**Published:** 2025-12-12

**Authors:** Joan Madia, Adrian A Boyle, James Ray, Alex Novak, Catherine J Pope, Bella Wheeler, Stavros Petrou, Raphael Wittenberg, Catia Nicodemo

**Affiliations:** 1 University of Oxford, Oxford, UK; 2 Addenbrooke’s Hospital Emergency Department, Cambridge, UK; 3 Emergency Department, Oxford University Hospitals NHS Foundation Trust, Oxford, UK; 4 Emergency Medicine Research Oxford (EMROx), Oxford University Hospitals NHS Foundation Trust, Oxford, UK; 5 Nuffield Department of Primary Care and Health Science, Oxford University, Oxford, UK; 6 Personal Social Services Research Unit, London School of Economics and Political Science, London, UK; 7 Nuffield Department of Primary Care Health Sciences, Oxford, UK; 8 Brunel University London, London, UK

**Keywords:** Health policy, Emergency Departments, Emergency Service, Hospital, Electronic Health Records, Health Services

## Abstract

**Abstract:**

**Objective:**

To examine how socioeconomic deprivation influences referral pathways to emergency departments (EDs) and to assess how these pathways affect subsequent hospital outcomes.

**Design:**

Retrospective observational study.

**Setting:**

Emergency department of a large teaching hospital in the East of England, providing secondary and tertiary care.

**Participants:**

482 787 ED attendances by patients aged 16 years and over, recorded between January 2019 and December 2023. Patients were assigned Index of Multiple Deprivation (IMD) deciles based on residential postcode.

**Main outcome measures:**

Referral source (general practitioner (GP), National Health Service (NHS) 111, ambulance, self-referral, other), total ED time, 4-hour breach, hospital admission and unplanned return within 72 hours.

**Results:**

Substantial socioeconomic inequalities were observed in referral pathways. Patients from the most deprived areas were significantly less likely to be referred by a GP (4.7%) than those from the least deprived areas (14.7%) and more likely to arrive via ambulance (32% vs 24%). These differences persisted after adjusting for demographic, clinical and contextual variables. Ambulance referrals showed the longest ED stays, ranging from 347 to 351 min across IMD deciles (overall 95% CI 343 to 363) and the highest probability of 4-hour breaches (51%; 95% CI 50% to 53%). Self-referrals had the greatest rates of unplanned returns within 7 days (up to 7.1%; 95% CI 5.5% to 8.7%). In contrast, NHS 111 and GP referrals were associated with shorter stays, lower breach rates and fewer reattendances. Minimal variation in outcomes was observed across deprivation levels once referral source was accounted for.

**Conclusions:**

Inequalities in how patients access emergency care, particularly reduced GP and NHS 111 referrals among more deprived groups, appear to underpin disparities in ED outcomes. Referral source captures important clinical and system-level factors that influence patient experience and resource use. Interventions to improve equitable access to structured referral pathways, particularly in more deprived areas, may enhance both the efficiency and fairness of emergency care delivery. Further research using national data is needed to assess broader policy implications and economic costs associated with differential access.

**STRENGTHS AND LIMITATIONS OF THIS STUDY**

- This study uses a large dataset of 482 787 emergency department attendances from a major National Health Service (NHS) teaching hospital, allowing robust analysis across socioeconomic groups.
- The inclusion of detailed referral source data, not available in national NHS datasets, enables a novel examination of pathways into emergency care.
- Adjustment for a wide range of demographic, clinical, temporal and system-level factors strengthens the validity of the findings.
- The analysis is observational, so causal relationships between deprivation, referral route and outcomes cannot be inferred.
- Findings are based on a single hospital in a relatively affluent region and may not fully generalise to more socioeconomically diverse or underserved populations.

## Introduction

 Emergency departments (EDs) are vital points of access within the healthcare system, yet persistent concerns remain about whether they are used equitably across different socioeconomic groups. In England, people living in more deprived areas are more likely to attend EDs, wait longer for care and experience poorer outcomes, including higher mortality rates.^1 2^ Understanding the mechanisms behind these inequalities is increasingly important, especially in the context of growing demand and efforts to streamline urgent care delivery within the National Health Service (NHS). The NHS is the publicly funded healthcare system of the UK, providing universal access to medical care free at the point of use. It is primarily financed through general taxation and operates under a gatekeeping model in which general practitioners (GPs) coordinate access to specialist and emergency services.

Research consistently shows a strong association between socioeconomic deprivation and increased ED utilisation. A study using national data found that ED attendance rates were approximately 0.45 per person per year in the least deprived areas and 0.68 per person per year in the most deprived areas, representing a 50% higher rate in the most deprived population,^3^ while another reported an OR of 1.69 for attendance in the most deprived decile.^4^ Patients from these areas are also more likely to experience longer waits,^5^ lower-acuity presentations^4^ and receive less complex care.^6^ International evidence supports similar trends, with disadvantaged populations in both the USA and Europe more likely to self-refer, face barriers to accessing primary care and be frequent users of emergency services.^7^

Less is known about how patients from deprived areas access ED services. Emerging evidence suggests that referral source and arrival mode—such as ambulance use or NHS 111 referrals—may play a role in structuring these inequalities.^7 8^ For example, studies have shown that lower-income groups are more likely to self-refer or arrive via ambulance, potentially bypassing earlier stages of care.^7^ Yet, much of this evidence originates from the USA, where insurance and payment mechanisms shape access differently from the UK’s NHS. In fact, the contribution of referral pathways to explaining socioeconomic disparities in ED outcomes remains poorly understood in the NHS setting.^9^,^11^ Yet few analyses have examined these pathways systematically within the NHS context, and only for the GP route,^10 12^ nor assessed how they influence downstream outcomes such as admission, length of stay or unplanned returns. Understanding how patients access emergency care—and how these pathways influence outcomes—is essential for designing equitable and efficient health systems.^7 13^

This study aims to examine whether and how socioeconomic deprivation influences referral pathways to EDs, and to assess how these pathways affect subsequent hospital outcomes. Specifically, we address two questions: first, are patients from more deprived areas more likely to arrive via ambulance or non-primary care routes? Second, does the referral source influence downstream outcomes such as admission, length of stay, 4-hour breaches or reattendance, and are these effects moderated by socioeconomic status?

To frame these questions, we draw on health services research and access-to-care theory, which together view referral pathways as mechanisms linking both the demand and supply sides of healthcare. From the demand perspective, patients’ choice or ability to access a referral route reflects differences in health literacy, socioeconomic barriers and timeliness of care-seeking. From the supply perspective, referral pathways reflect prior triage, diagnostic preparation and coordination across services; elements that influence how efficiently patients are processed once they reach the ED. Clinically, we acknowledge that doctors prioritise treatment based on the severity of illness, not on referral route; however, referral source shapes *when* and *how* patients arrive, and the extent to which clinical information accompanies them. Patients referred by GPs or NHS 111 often arrive earlier in their disease course, with clearer documentation and pre-triage assessments, whereas self-referrals or ambulance arrivals may present with delayed or undifferentiated symptoms. These differences can produce variation in downstream outcomes such as waiting times, admissions or reattendance, not because of unequal treatment, but because of differing pathways and system interactions preceding ED entry.

## Methods

### Data and participants

We conducted a retrospective observational study using routinely collected data from Addenbrooke’s Hospital, part of Cambridge University Hospitals (CUH) NHS foundation trust. CUH is a large teaching hospital located in Cambridge, East of England, serving both the city and the surrounding counties. The catchment area includes populations with varying levels of deprivation but is, on average, less deprived than the national mean. While CUH’s patient population is not fully representative of England as a whole, it provides a diverse mix of urban, suburban and rural patients, offering valuable insights into referral behaviours across different socioeconomic contexts. Further details on the hospital’s catchment characteristics and representativeness are provided in onlinesupplemental figures A6  A7.

The sample covers the period from January 2019 to December 2023. This period includes the COVID-19 pandemic (2020–2022), which substantially affected ED utilisation patterns and referral behaviours across the NHS. To account for this, we included year and month fixed effects in all models to control for temporal shocks related to the pandemic, ensuring that our estimated associations reflect broader patterns rather than pandemic-specific fluctuations. The dataset includes all ED attendances by patients aged 16 and over. Each attendance was treated as a separate observation, as the unit of analysis was the ED visit rather than the individual patient. Some patients attended more than once during the study period, and we accounted for this non-independence by using robust SEs clustered at the patient level in all models.

Attendances by children and adolescents (≤15 years) were excluded (117 593 attendances, 18.8%), as they are managed in a separate paediatric unit. We also excluded 43 cases from 2018 (due to partial data, 0.01%), 4 cases in which patients were recorded as dead on arrival (0.001%) and 25 921 cases with missing values in key variables (4.1%). The final analytical sample comprised 482 787 ED visits (77.1% of the initial dataset). A flow diagram illustrating the construction of the analytical sample is provided in online supplemental figure A1.

### Covariates

We examined a range of variables to explore how socioeconomic deprivation and referral source jointly influence both attendance to the ED and subsequent service performance outcomes, such as length of stay, admission and reattendance. The main explanatory variable was socioeconomic deprivation, measured using the Index of Multiple Deprivation (IMD).

In the first stage of analysis, the referral source was treated as an outcome variable, used to examine whether patterns of access to the ED differed across IMD deciles. In the second stage, the referral source was treated as a key explanatory variable in models of downstream outcomes. In these models, we included dummy variables for all ten IMD deciles and all six referral categories (GP, NHS 111, ambulance, self-referral, other medical and other non-medical), as well as interaction terms between IMD and referral source (IMD×referral) to capture whether the effect of referral route varied by socioeconomic status. This two-stage design allowed us to assess both inequality in access pathways and the implications of those pathways for subsequent hospital outcomes. We then considered the referral source as another key explanatory factor. In an initial stage of the analysis, the referral source itself was treated as an outcome to examine variation in access routes.

Unlike standard NHS Digital datasets such as the Emergency Care Data Set (ECDS) within Hospital Episode Statistics, this information was available in our data, allowing a more detailed investigation. In addition, we included a wide range of covariates. Socio-demographic factors included gender (categorised as male or female), age group (categorised from 16 to 24 to 85+) and UK residency status. Clinical acuity was measured using the first recorded National Early Warning Score (NEWS), a validated physiological scoring system that uses vital signs to identify patients at risk of clinical deterioration.^14^ NHS datasets, by contrast, typically report acuity in aggregated categories, whereas our data retain the full score, allowing a more detailed assessment of patient condition. Reason for attendance was grouped into diagnostic categories including trauma and injuries, cardiovascular conditions, respiratory conditions, gastrointestinal problems, neurological conditions, psychiatric presentations and other medical conditions (see online supplemental materials section 1), the arrival mode (ambulance, walk-in, etc) as an access-related outcome and ED treatment area captured the initial area of care (minors; majors with patients either ambulant or trolley; or resuscitation).

To capture variation in ED operational context, we included shift type (day vs night), day of the week and calendar month and year. Workload and system pressure were proxied by the total number of ED attendances and admissions per day, month and year, as well as a 3-day rolling average of arrivals. Finally, patient residence was grouped into 13 geographical categories to reflect the catchment area of the CUH, a large teaching hospital and major trauma centre serving both local and regional populations. These categories included Cambridge city, surrounding Cambridgeshire districts and adjacent counties such as Suffolk, Norfolk and Essex, as well as areas further afield, including London and the rest of England.

The primary hospital outcomes were: (1) total time spent in the ED (measured continuously, in minutes from arrival to departure); (2) whether the patient remained in the ED for more than 4 hours (binary variable; the 4-hour threshold is a national NHS target that aims for 95% of ED patients to be admitted, transferred or discharged within 4 hours); (3) whether the patient was admitted to hospital following ED attendance (binary variable); and (4) whether the patient made an unplanned return within 72 hours (binary variable). The 4-hour threshold is a national performance target in the UK’s NHS, which aims for at least 95% of ED patients to be admitted, transferred or discharged within 4 hours of arrival. It is a key indicator of hospital flow and service efficiency.

### Statistical analysis

The analysis followed two stages aligned with the study aims. First, logistic regression models examined how socioeconomic deprivation (measured by IMD deciles) predicted referral source (GP, NHS 111, ambulance, self-referral, other medical and other non-medical).


$$logit(Pr(Yi=k))=\beta 0+\beta 1IMDi+\beta 2Xi^{\prime }+\epsilon i$$

Yi denotes the referral source category for patient i (GP, NHS 111, ambulance, self-referral, other medical or other non-medical), IMDi represents the IMD in deciles, Xi′ is a vector of covariates including demographic, clinical, temporal and contextual variables, and εi is the error term.

This stage assessed whether patterns of access to the ED varied by socioeconomic status. A conceptual framework outlining the assumed relationships among deprivation, referral source, outcomes and covariates is presented in online supplemental figure A2 (directed acyclic graph). This diagram clarifies which variables were treated as potential confounders and which were included as contextual factors to account for system-level variation.

Second, we examined how referral source and deprivation jointly influenced downstream hospital outcomes: total ED time (continuous), 4-hour breach (binary), hospital admission (binary) and unplanned return within 72 hours (binary). To capture potential moderation effects, we included interaction terms between IMD deciles and referral source (IMD×referral source) in each outcome model. These interaction terms tested whether the relationship between referral pathway and outcomes differed across socioeconomic groups.


$$Yi=\begin{array}{l} \alpha 0+\alpha 1\;IMDi+\alpha 2\;Referrali+\\ \alpha 3\;(IMDi\times Referrali)+\alpha 4\;Xi\prime +\epsilon i \end{array}$$

Where Yi represents the hospital outcome for attendance—namely, total ED time, 4-hour breach, hospital admission or unplanned return within 72 hours. α1 IMDi is the IMD decile, (IMDi×Referrali) denotes the referral source category to capture the interaction effects, testing whether the impact of referral source varies by deprivation level, Xi′ is a vector of control variables (demographics, clinical acuity, attendance reason, arrival mode, treatment area, time effects and workload indicators), and εi is the error term.

Binary outcomes were estimated using logistic regression, and results are presented as average marginal effects (AMEs). AMEs express the average change in the probability of the outcome associated with a one-unit change in each predictor, holding other variables constant. Unlike ORs, AMEs are reported on the probability scale, making them directly interpretable and comparable across models. For the continuous outcome of total ED time, we applied a generalised linear model with a log link and gamma family, chosen after assessing non-normality using residual plots and the modified Park test. All models adjusted for patient demographics, clinical acuity (NEWS), attendance reason, arrival mode, treatment area, time effects and workload indicators. Robust SEs were clustered by patient to account for repeat attendances. Analyses were performed in Stata V.17 (StataCorp LLC, College Station, Texas, USA).

### Patient and public involvement

Patients and members of the public were involved in this study from its conception through dissemination. Our patient advisory group, comprising individuals with lived experience of ED use from diverse socioeconomic backgrounds, contributed to: (1) refining the research questions to ensure they addressed patient priorities; (2) reviewing the study protocol and data collection processes; (3) interpreting findings through dedicated workshops; and (4) shaping the dissemination strategy to maximise impact for service users.

## Results

The analytical sample comprised 482 787 ED visits between January 2019 and December 2023. Women accounted for just over half of all attendances (53%). The age distribution was relatively even across working-age groups, with the largest proportions aged 25–34 years (16%) and 16–24 years (15%), while 21% of visits involved patients aged 75 years or older. Most attendances (41%) were self-referrals, followed by ambulance referrals (27%) and those from GPs or practice nurses (13%). Consistent with these referral patterns, around 69% of patients arrived by walking in and 29% by emergency road ambulance. Attendance showed some variation by deprivation level, with higher proportions of visits from residents in less deprived areas (IMD deciles 7–10, 64%) than from the most deprived areas (deciles 1–3, 8%). Many attendances were from residents in Cambridge and South Cambridgeshire (58%), with smaller shares from neighbouring counties such as Suffolk and Essex (see online supplemental table A1).

### Attendance route to the emergency department by deprivation

**Figure 1:**
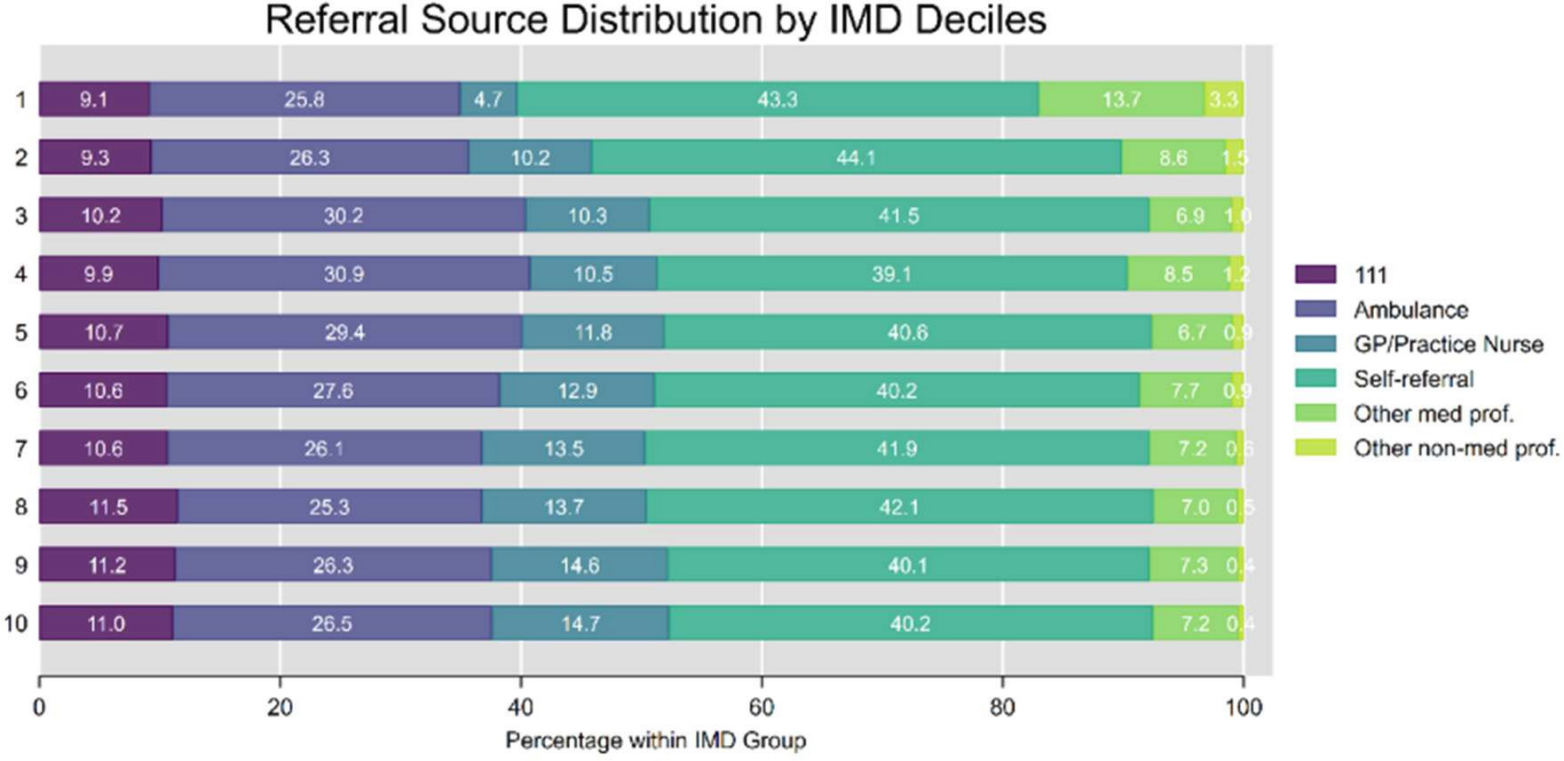
Referral source distribution by IMD deciles. GP, general practitioner; IMD, Index of Multiple Deprivation.

**Figure 2:**
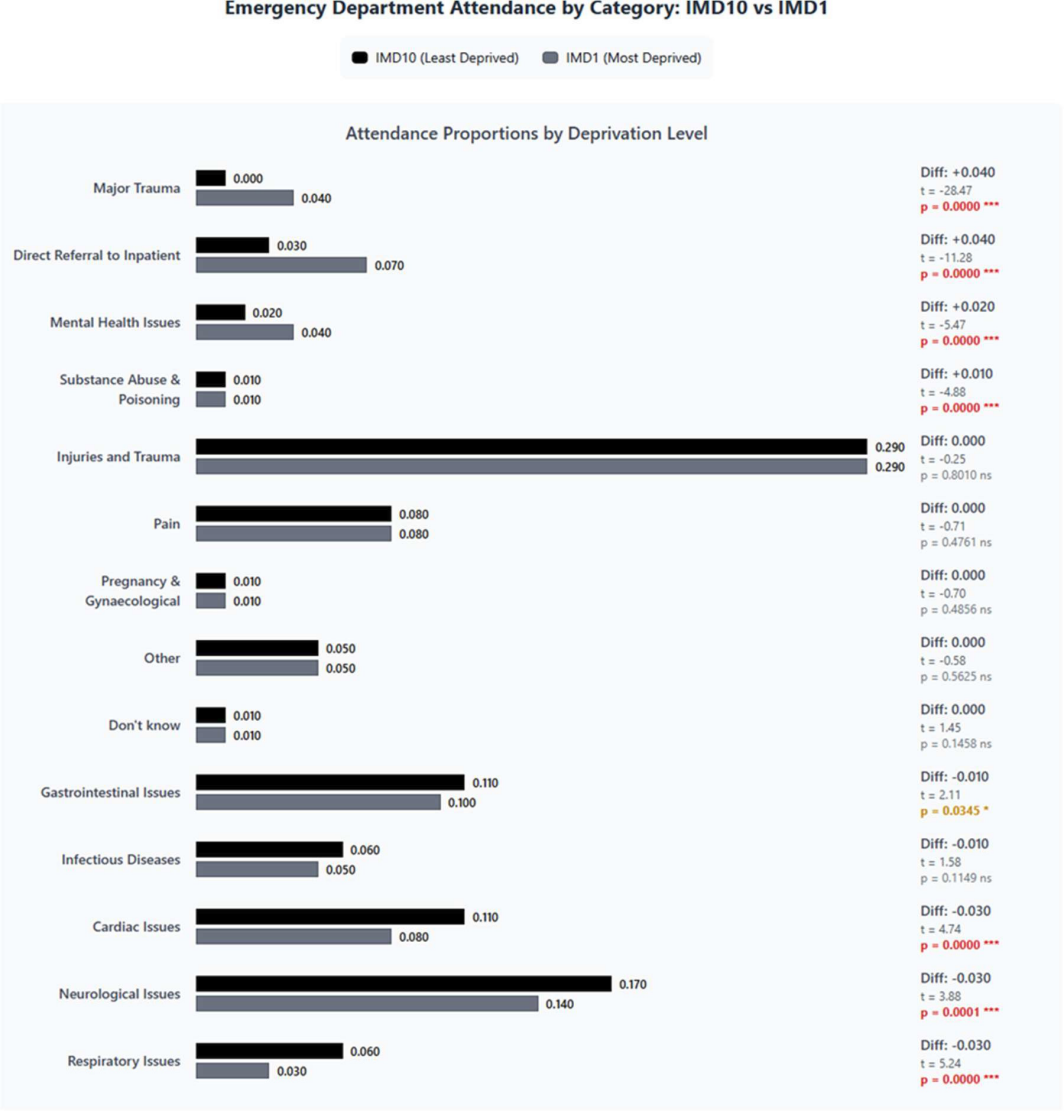
Attendance reasons for IMD10 (least deprived) and IMD1 (most deprived). IMD, Index of Multiple Deprivation.

Figure 1 presents the distribution of referral sources across the IMD in deciles. There were marked differences in how patients accessed ED services. Self-referral was the predominant pathway across all IMD deciles (39–44%), though slightly higher in the most deprived areas. A striking social gradient was observed in GP/practitioner referrals, with rates more than tripling from the most deprived (4.71%) to the least deprived areas (14.69%). NHS 111 service referral also showed a modest social gradient, increasing gradually from the most deprived areas (9.13%) to less deprived areas (peaking at 11.48% in decile 8). Ambulance utilisation was highest in middle-deprivation areas (approximately 30% in deciles 3–4) compared with both extremes of the deprivation spectrum. Further analysis revealed that ‘Other medical’ referrals primarily consisting of advanced care practitioners, outpatient clinics and EDs were substantially higher in the most deprived areas (13.74% in decile 1) compared with less deprived areas (around 7% in deciles 5–10). This may reflect the presence of additional community-based services in more deprived areas, such as urgent care centres or walk-in clinics, that are organisationally linked to local GP practices and capable of referring patients to the ED. Similarly, ‘Other non-medical’ referrals, predominantly from police/forensic medical officers, showed a clear deprivation gradient, decreasing from 3.25% in the most deprived areas to just 0.40% in the least deprived areas. These patterns highlight significant socioeconomic inequalities in emergency care access pathways. This could be explained by the fact that the people from deprived areas need a different type of care than primary care due to injuries and trauma and mental health acuity, as we can see in figure 2. Onlinesupplemental figures A8  A9 show the distribution of deprivation within referral sources and attendance reasons, offering a complementary perspective on which patient groups are over-represented in specific pathways or presentations.

**Figure 3:**
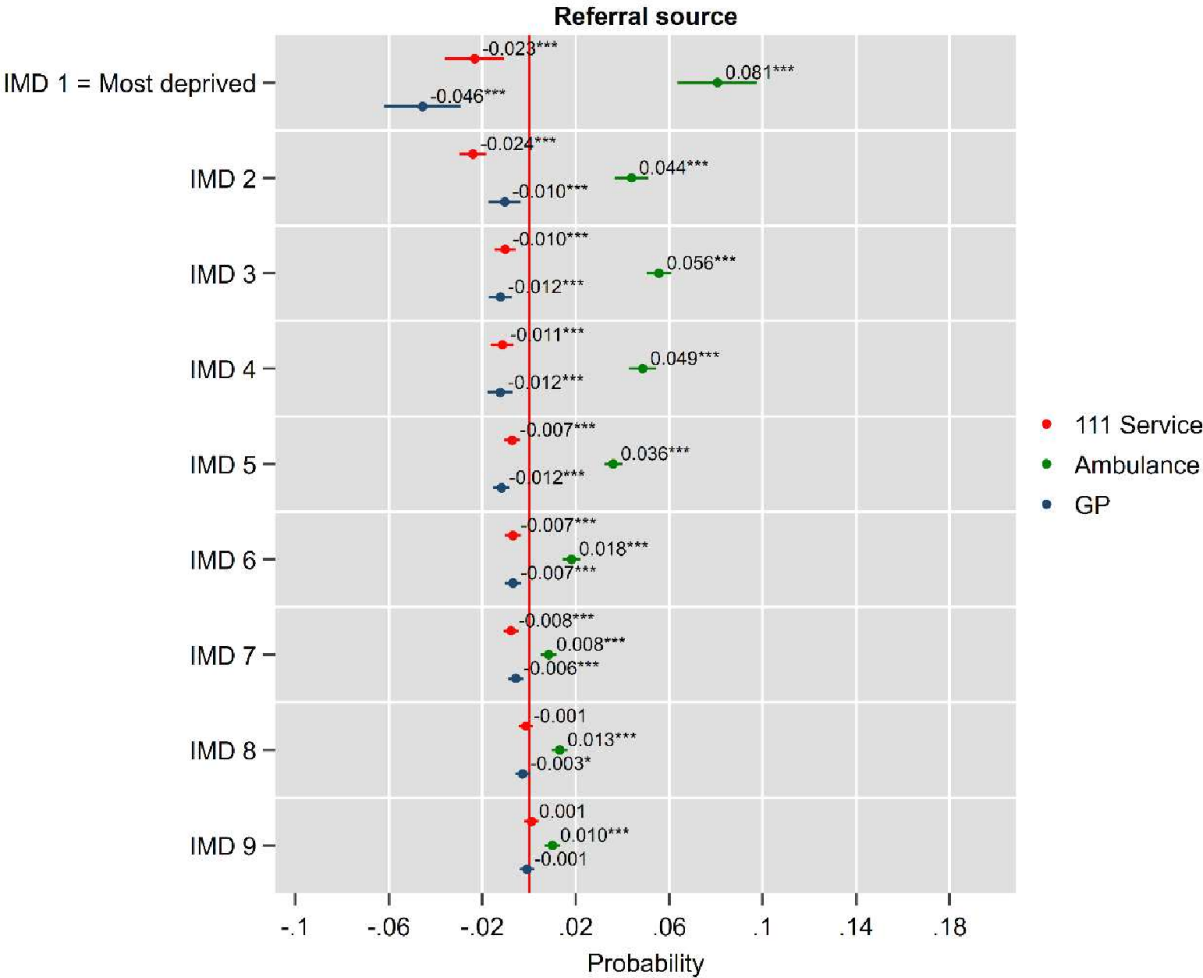
AME on probability of being referred by 111, ambulance and GP. AME, average marginal effects; GP, general practitioner; IMD, Index of Multiple Deprivation.

Figure 3 presents average marginal effects from binary logistic regression models, estimating the probability of referral to the ED via NHS 111, ambulance or GP, expressed as probability differences (results for all the referral sources in the online supplemental table A2). The most striking finding is the clear socioeconomic gradient in GP referrals, with significantly lower probabilities of GP referrals in more deprived areas compared with the reference group (IMD 10, least deprived). This gradient persists even after adjusting for socio-demographic, attendance reasons, clinical and temporal covariates, with the most deprived areas (IMD 1) a 4.6 percentage point lower probability of admission compared with those from the least deprived areas (p<0.001). For the 111 service, we observe significant negative effects for most IMD deciles relative to the least deprived areas, with the strongest effects in the most deprived areas (IMD 1: 2.8 percentage points lower, p<0.001), though this gradient becomes less pronounced in middle deprivation areas. Conversely, ambulance referrals show a positive association with deprivation, with significantly higher probabilities in more deprived areas compared with the least deprived, particularly in IMD 1–4 (ranging from 8.1 to 4.9 percentage points higher, p<0.001). These findings demonstrate that socioeconomic inequalities in ED referral pathways persist even after accounting for comprehensive clinical, demographic and contextual factors.^10 11^ Specifically, patients in IMD 1 were more likely to arrive via ambulance, less likely to be referred by NHS 111 and less likely to be referred by a GP compared with those in IMD 10. Similar patterns for ambulance arrivals are observed when considering arrival mode, even after adjusting for demographics, attendance reasons and clinical acuity (online supplemental figure A3).

### Deprivation, referral source and ED outcomes

**Figure 4:**
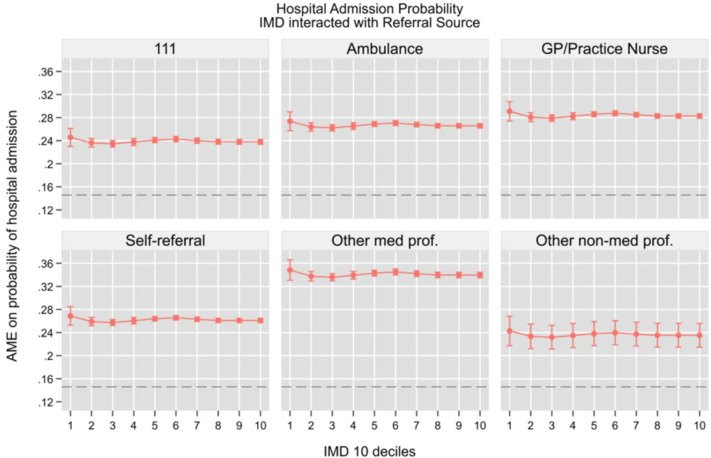
Hospital admission probability, AME from IMD and referral source interaction. Note: The horizontal dashed line represents the overall average marginal effect (0.15). All models control for socio-demographic characteristics, area of residence, NEWS 2 score, attendance category, hospital area (majors, minors, resuscitation, paediatric), COVID-19 isolation status, day of the week, month, year, whether the attendance occurred during a night shift and system-pressure measures including the total number of daily ED arrivals, daily emergency admissions and the 3-day rolling average of arrivals, which serve as proxies for crowding and workload. AME, average marginal effects; GP, general practitioner; IMD, Index of Multiple Deprivation; NEWS, National Early Warning Score.

**Figure 5:**
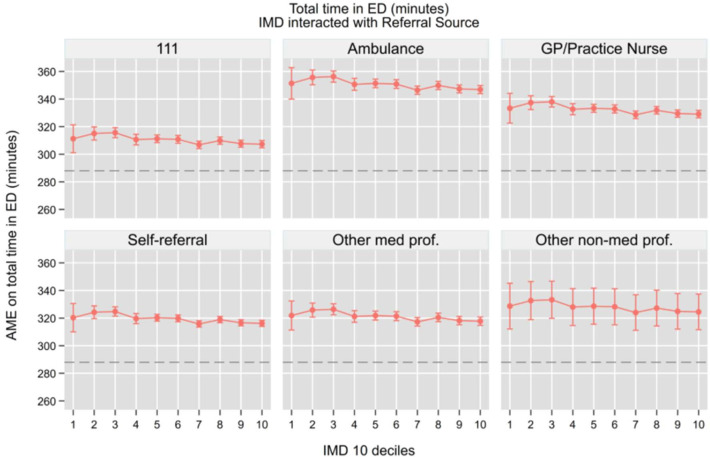
Total time in ED (minutes), AME from IMD and referral source interaction. Note: The horizontal dashed line represents the overall average marginal effect (288). All models control for socio-demographic characteristics, area of residence, NEWS 2 score, attendance category, hospital area (majors, minors, resuscitation, paediatric), COVID-19 isolation status, day of the week, month, year, whether the attendance occurred during a night shift and system-pressure measures including the total number of daily ED arrivals, daily emergency admissions and the 3-day rolling average of arrivals, which serve as proxies for crowding and workload. AME, average marginal effects; ED, emergency department; GP, general practitioner; IMD, Index of Multiple Deprivation; NEWS, National Early Warning Score.

**Figure 6:**
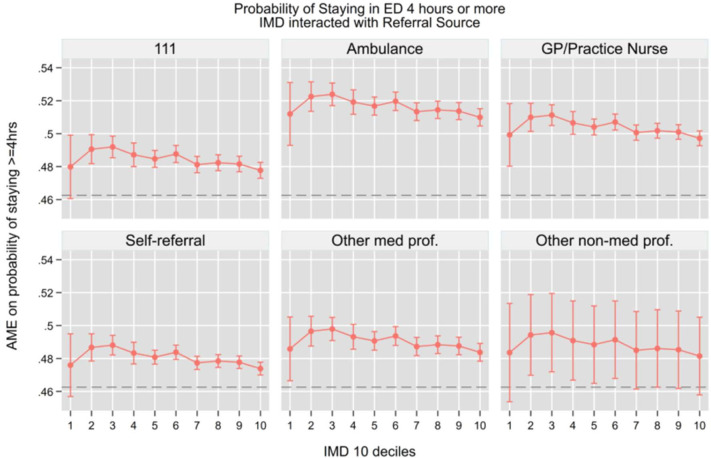
Probability of staying in ED 4 hours or more. Note: The horizontal dashed line represents the overall average marginal effect (0.463). All models control for socio-demographic characteristics, area of residence, NEWS 2 score, attendance category, hospital area (majors, minors, resuscitation, paediatric), COVID-19 isolation status, day of the week, month, year, whether the attendance occurred during a night shift and system-pressure measures including the total number of daily ED arrivals, daily emergency admissions and the 3-day rolling average of arrivals, which serve as proxies for crowding and workload. AME, average marginal effects; ED, emergency department; GP, general practitioner; IMD, Index of Multiple Deprivation; NEWS, National Early Warning Score.

**Figure 7:**
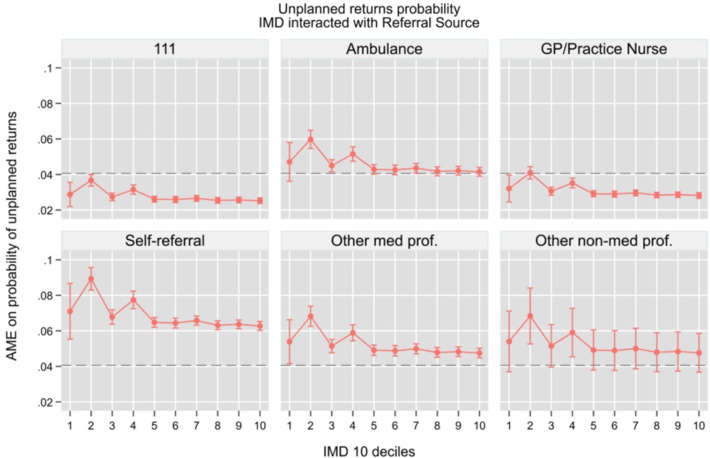
Unplanned returns to ED, AME from IMD and referral source interaction. Note: The horizontal dashed line represents the overall average marginal effect (0.04). All models control for socio-demographic characteristics, area of residence, NEWS 2 score, attendance category, hospital area (majors, minors, resuscitation, paediatric), COVID-19 isolation status, day of the week, month, year, whether the attendance occurred during a night shift and system-pressure measures including the total number of daily ED arrivals, daily emergency admissions and the 3-day rolling average of arrivals, which serve as proxies for crowding and workload. AME, average marginal effects; ED, emergency department; GP, general practitioner; IMD, Index of Multiple Deprivation; NEWS, National Early Warning Score.

Building on our previous analysis of ED access patterns, we now examine how referral sources interact with socioeconomic status to influence downstream hospital outcomes. Using similar regression models but now including interaction terms between IMD deciles and referral sources, we analysed four key outcomes while controlling for the same comprehensive set of covariates: hospital admission (be admitted in the hospital as an inpatient), total time in department, 4-hour breaches and unplanned returns to the ED. We summarise the key findings graphically in the main figures 4–7, while the full set of AMEs from the interaction models is provided in the online supplemental tables A3–A6. This analysis allows us to understand how the initial pathway into emergency care shapes subsequent clinical pathways and resource utilisation across different socioeconomic groups.

Our findings reveal that referral source was substantially more influential than socioeconomic status in determining hospital service performance outcomes, a pattern already observed when considering unconditional trends over time (online supplemental figure A4). For hospital admissions (value 1 if the patient is admitted as an inpatient and 0 otherwise), see figure 4. Patients referred by other medical professionals consistently had the highest adjusted admission probabilities (34.9–36.4%, p<0.001) across all deprivation levels, with remarkably little variation by IMD decile. GP referrals also showed relatively high admission rates (28.9–30.2%, p<0.001), exhibiting only a modest socioeconomic gradient that was not clinically significant.

In contrast, NHS 111 referrals demonstrated consistently lower admission probabilities (24.1–25.3%, p<0.001) across all IMD groups. Self-referrals and ambulance arrivals occupied an intermediate position, with admission probabilities clustering around 26–27% (p<0.001) and showing no meaningful socioeconomic pattern. Notably, within each referral type, differences across deprivation levels were generally small (1–2 percentage points) with overlapping CIs, suggesting that the referral pathway conveys meaningful clinical or triage information that influences admission decisions independently of patients’ socioeconomic background.

Total time in department (figure 5) showed significant variation by referral source but minimal differences across the deprivation spectrum. Patients arriving by ambulance experienced the longest adjusted length of stay (347–351 min, p<0.001), regardless of deprivation, with only a marginal decrease in less deprived areas. GP referrals were also associated with extended ED stays (329–333 min, p<0.001). In striking contrast, patients referred through NHS 111 consistently spent the least amount of time in ED (307–311 min, p<0.001), representing a clinically meaningful difference of 40–45 min compared with ambulance arrivals. Self-referrals showed intermediate stay times (316–320 min, p<0.001) with minimal variation across IMD deciles. While there was a slight gradient showing shorter ED stays in less deprived areas, these differences were relatively modest (5–10 min) and clinically negligible compared with the substantial differences observed between referral sources.

Similarly, for 4-hour breaches (figure 6), the probability of exceeding the target time was predominantly associated with the referral pathway rather than socioeconomic status. Ambulance arrivals consistently had the highest probability of breaching the 4-hour target (50.9–51.2%, p<0.001) across all IMD deciles, closely followed by GP-referred patients (49.7–49.9%, p<0.001). Self-referrals and NHS 111 referrals demonstrated lower breach probabilities (47.4–47.9%, p<0.001). All referral types showed flat or slightly decreasing trends from most to least deprived deciles, but these differences were small (1–2 percentage points) and not clinically significant, reinforcing that referral source, likely reflecting differences in triage acuity, prioritisation, need for hospital admission or clinical complexity, was more predictive of ED processing time than socioeconomic background. Interestingly, we also notice in online supplemental figure 5A that admission probability rises steadily with time in the ED, peaking sharply around 240 min, suggesting increased decision pressure near the NHS 4-hour target.

Unplanned returns within 7 days (figure 7) revealed a more complex pattern, with both referral source and deprivation playing important roles. Self-referrals had significantly higher probabilities of return across all IMD deciles (6.3–7.1%, p<0.001), with a notable socioeconomic gradient showing higher rates in more deprived areas. Patients arriving by ambulance or referred by other medical professionals also had moderately high return probabilities within 72 hours (4.1–4.7% and 4.8–5.4%, respectively, p<0.001). In contrast, NHS 111 and GP referrals demonstrated substantially lower reattendance rates (2.5–2.9% and 2.8–3.2%, respectively, p<0.001), suggesting that structured, formal referrals were associated with more appropriate and effective care episodes. The socioeconomic gradient was consistent across all referral types, with deprived areas showing higher unplanned return rates, though the magnitude of difference between referral sources exceeded the variation attributable to deprivation alone also this effect persisted after controlling for acuity and attendance reasons of the patients.

## Discussion

Overall, our findings reveal clear socioeconomic disparities in emergency care access and outcomes, suggesting that much of the inequality arises before patients even reach the ED. Deprived populations were significantly less likely to be referred by GPs and via NHS 111, and more likely to arrive via ambulance, even after accounting for attendance reasons, socio-demographic, clinical and temporal covariates. However, referral source emerged as the primary determinant of subsequent ED outcomes, including admissions, unplanned returns, 4-hour breaches and total time in ED. This suggests that much of the observed inequality in hospital service performance is shaped before ED presentation, particularly through differential access to and engagement with primary or urgent care services.

In some cases, patients from more deprived areas may arrive at the ED with undiagnosed or unmanaged conditions due to gaps in earlier care, requiring more extensive investigations or treatment. As such, longer stays and higher admission rates may reflect delayed presentations or missed opportunities for earlier intervention, rather than acute severity at the point of triage alone. This interpretation aligns with qualitative findings indicating that EDs increasingly serve as a safety net for unmet healthcare needs, particularly among socioeconomically disadvantaged groups. Patients from more deprived areas are more likely to access the ED via ambulance or self-presentation, and less likely to arrive following a referral from general practice or NHS 111. This pattern is consistent with evidence showing that some patients attend EDs because they are unable to access primary care services, with avoidable ED attendance appearing to be mostly driven by underlying deprivation rather than by the degree of access to primary care.^10 15 16^ While we cannot observe upstream triage decisions or those who were advised to self-manage, these patterns suggest differences in how socioeconomic groups interact with or are supported by the broader urgent and primary care system. This reliance on unplanned and emergency routes is compounded by higher rates of police involvement and referrals from other medical professionals, pointing to broader social and clinical vulnerabilities. In contrast, less deprived populations appear to make more use of primary care and urgent care system-level triage, enabling more structured and potentially more appropriate referrals into the ED. Together, these findings point to underlying inequities in how different socioeconomic groups navigate and are supported by the primary, urgent and emergency care system.

However, once patients enter the department, referral source, rather than socioeconomic status, emerges as the predominant driver of outcomes. People from deprived areas indeed have more injuries, trauma and acute mental health issues, which could justify the use of the most expensive care service, such as the ambulance; however, after controlling for these variables, such as reason of attendance and acuity, we still observe an overuse of these services that is not explained by these factors. This suggests a two-stage process of inequality: first, in the pathways to emergency care, and second, in the subsequent care experience shaped by these modes of entry.

The striking threefold difference in GP referrals between the least and most deprived areas (14.69% vs 4.71%) likely reflects broader structural inequalities in primary care access. These may be driven by supply side (limited GP availability, appointment barriers) and demand side barriers (lower healthcare literacy and reduced trust in healthcare institutions) in more deprived areas. The financial impact of these inequalities is significant, particularly as ambulance services are costly.

In 2023/2024, the average cost of transporting a patient to accident and emergency department by ambulance was £459, while ambulance callouts that did not result in a hospital trip cost an average of £327.^9^ In contrast, accessing care via NHS 111 is considerably less expensive. Importantly, the downstream consequences of these access inequalities are substantial. Patients referred through primary or urgent care pathways, such as GP or NHS 111, consistently demonstrated better waiting time outcomes (shorter ED stays, fewer 4-hour breaches), lower admission rates and markedly lower rates of unplanned returns compared with those who self-referred or arrived via ambulance. These patterns persisted after adjusting for a wide range of demographic and clinical factors, suggesting that referral source may also capture underlying differences in health-seeking behaviours, preferences and patterns of service use across socioeconomic groups; factors not fully reflected in clinical acuity scores. The consistently lower unplanned return rates among GP and 111 referrals in particular highlight the value of effective triage and care navigation before ED attendance.

It is important to emphasise that these are observational associations, not causal estimates. While we adjusted for known confounders, unmeasured factors may still influence both referral pathways and outcomes. Moreover, our data come from the CUH, a large teaching hospital located in one of the more affluent regions of England. As such, findings regarding the IMD may not generalise to more socioeconomically diverse or underserved areas and should be interpreted in that context. However, a key strength of this dataset lies in its detailed information on referral source, linkage to IMD and the inclusion of NEWS, providing a more accurate measure of patient acuity than is currently available in many versions of the NHS datasets like ECDS. In this sense, our findings also underscore the need for improved and standardised national data to better understand inequality patterns in secondary care.

Overall, our findings suggest that policy efforts should focus on two complementary areas. First, addressing socioeconomic inequalities in how patients access and engage with primary and urgent care services, including barriers to GP use and limited uptake of NHS 111, may help reduce disparities in ED referral patterns. Second, there is scope to improve the effectiveness and follow-up of self-referral pathways, which remain the predominant route across all deprivation levels yet are associated with higher reattendance and poorer outcomes.

While part of the variation in outcomes may reflect differences in underlying acuity or triage effectiveness, particularly in the case of NHS 111 referrals, the consistently lower unplanned return rates for patients entering via structured pathways point to the potential benefits of earlier, more appropriate engagement with care. Strengthening NHS 111 services, and supporting their use in more deprived areas, may therefore be a promising strategy, especially if combined with measures to ensure equitable access and trust in non-emergency services. Together, such approaches could help improve the appropriateness, efficiency and equity of emergency care delivery. Future research should aim to replicate these findings using nationally representative data, further investigate the mechanisms behind differential access by deprivation level, and assess the potential cost implications for the NHS of these unequal care pathways.

## Supplementary material

10.1136/bmjopen-2025-108770 Supplementary Figure 1

10.1136/bmjopen-2025-108770 Supplementary Figure 2

10.1136/bmjopen-2025-108770 Supplementary Figure 3

10.1136/bmjopen-2025-108770 Supplementary Figure 4

10.1136/bmjopen-2025-108770 Supplementary Figure 5

10.1136/bmjopen-2025-108770 Supplementary Figure 6

10.1136/bmjopen-2025-108770 Supplementary Figure 7

10.1136/bmjopen-2025-108770 Supplementary Figure 8

10.1136/bmjopen-2025-108770 Supplementary Figure 9

10.1136/bmjopen-2025-108770 Supplementary file 1

## Data Availability

Data may be obtained from a third party and are not publicly available.
