# Supplementary figures and images for: Socioeconomic inequality and access to emergency care: understanding the pathways to the emergency department in the UK
Source: BMJ Open. 2025 Dec 12;15(12):e108770. doi: 10.1136/bmjopen-2025-108770 (PMC12706212; doi:10.1136/bmjopen-2025-108770)

Figure A.1: Development of analytical dataset

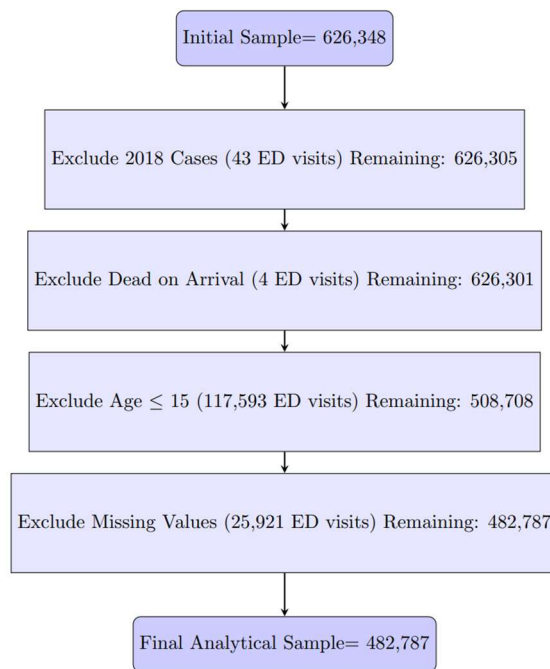

Supplement: Supplementary Figure 1 [file bmjopen-15-12-s001.pdf]

Figure A.2: Conceptual framework linking deprivation, referral source, and hospital outcomes.

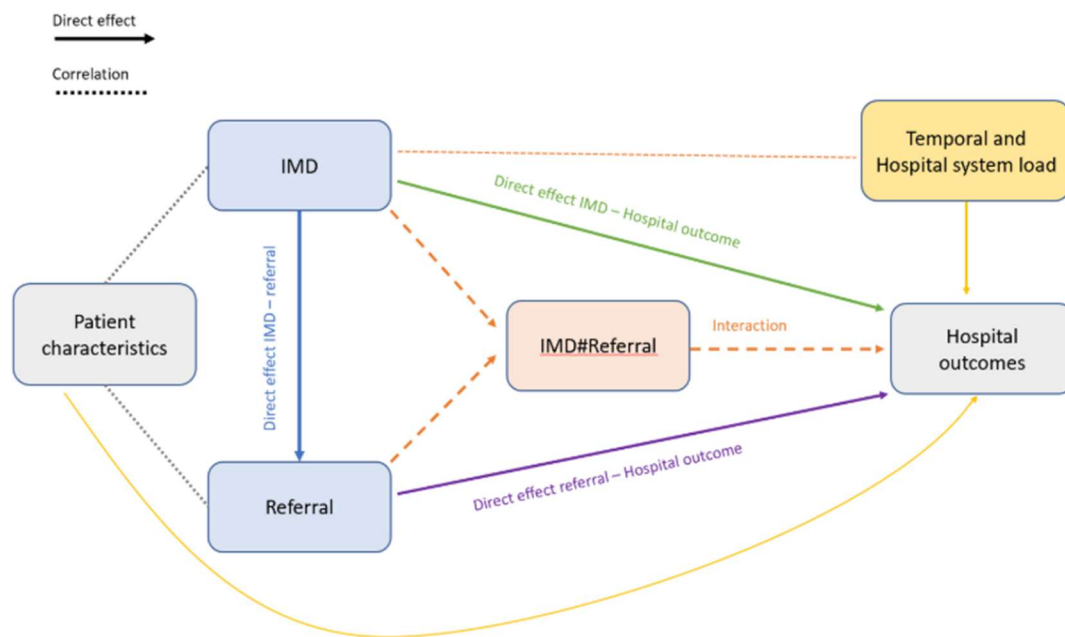

Supplement: Supplementary Figure 2 [file bmjopen-15-12-s002.pdf]

Figure A.6: Overall Absolute distribution of IMD deciles

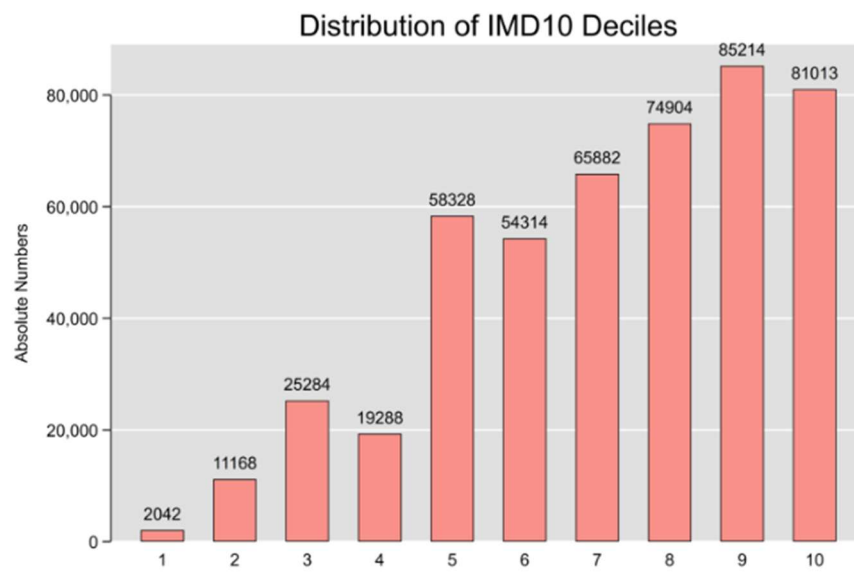

Supplement: Supplementary Figure 6 [file bmjopen-15-12-s006.pdf]

Figure A.7: Distribution of Catchment areas by Year

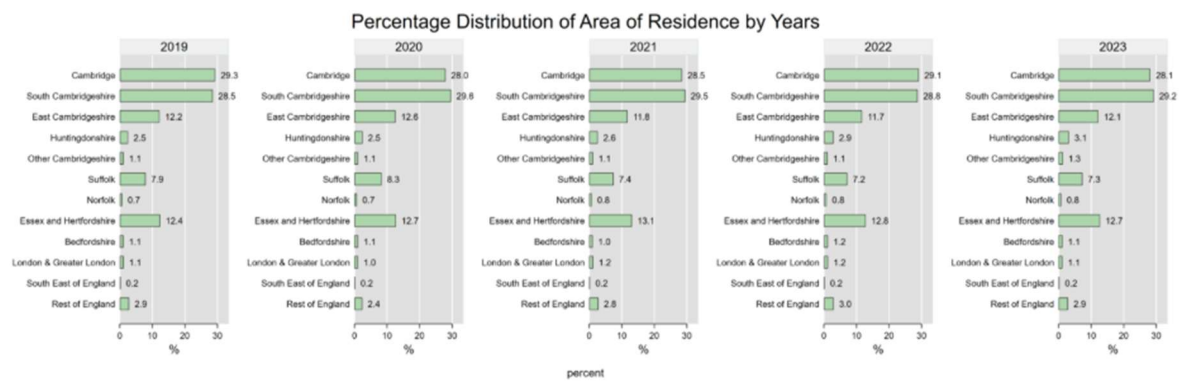

Supplement: Supplementary Figure 7 [file bmjopen-15-12-s007.pdf]

Figure A.8: IMD Decile Distribution by Referral Source

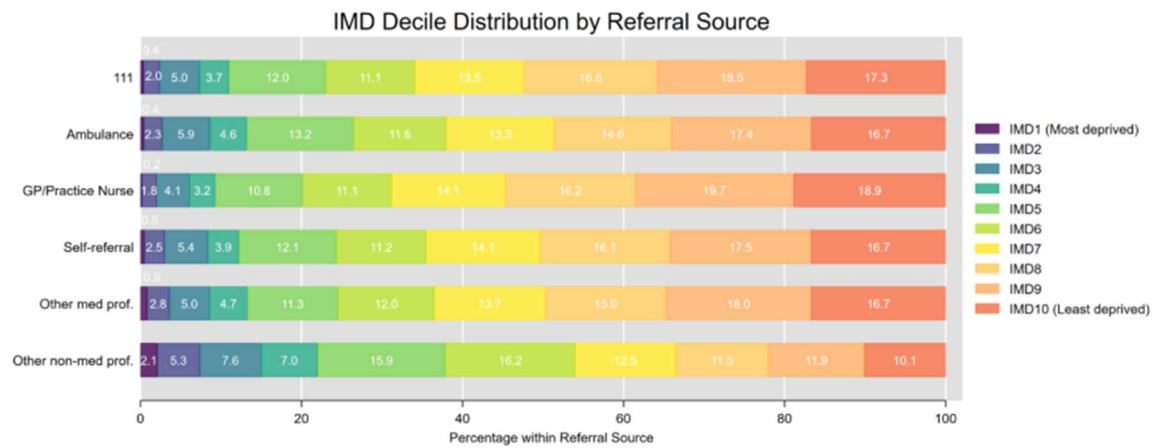

Supplement: Supplementary Figure 8 [file bmjopen-15-12-s008.pdf]

Figure A.9: IMD Decile Distribution by Attendance Category

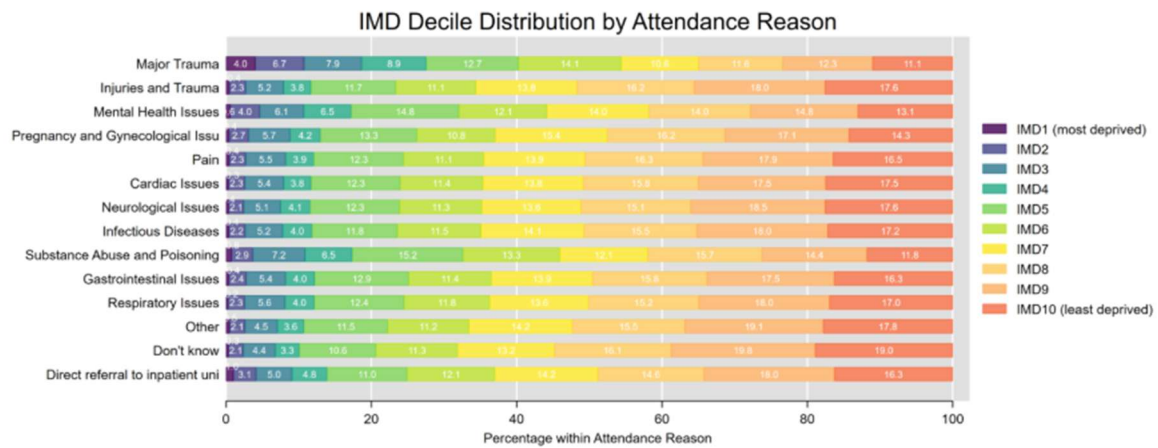

Supplement: Supplementary Figure 9 [file bmjopen-15-12-s009.pdf]
