# Supplementary material for: Socioeconomic inequality and access to emergency care: understanding the pathways to the emergency department in the UK
Source: BMJ Open. 2025 Dec 12;15(12):e108770. doi: 10.1136/bmjopen-2025-108770 (PMC12706212; doi:10.1136/bmjopen-2025-108770)

Figure A.3: Probability of Arriving to the ED in an emergency road ambulance, AME, Additional results on Arrival mode: Emergency Road Ambulance

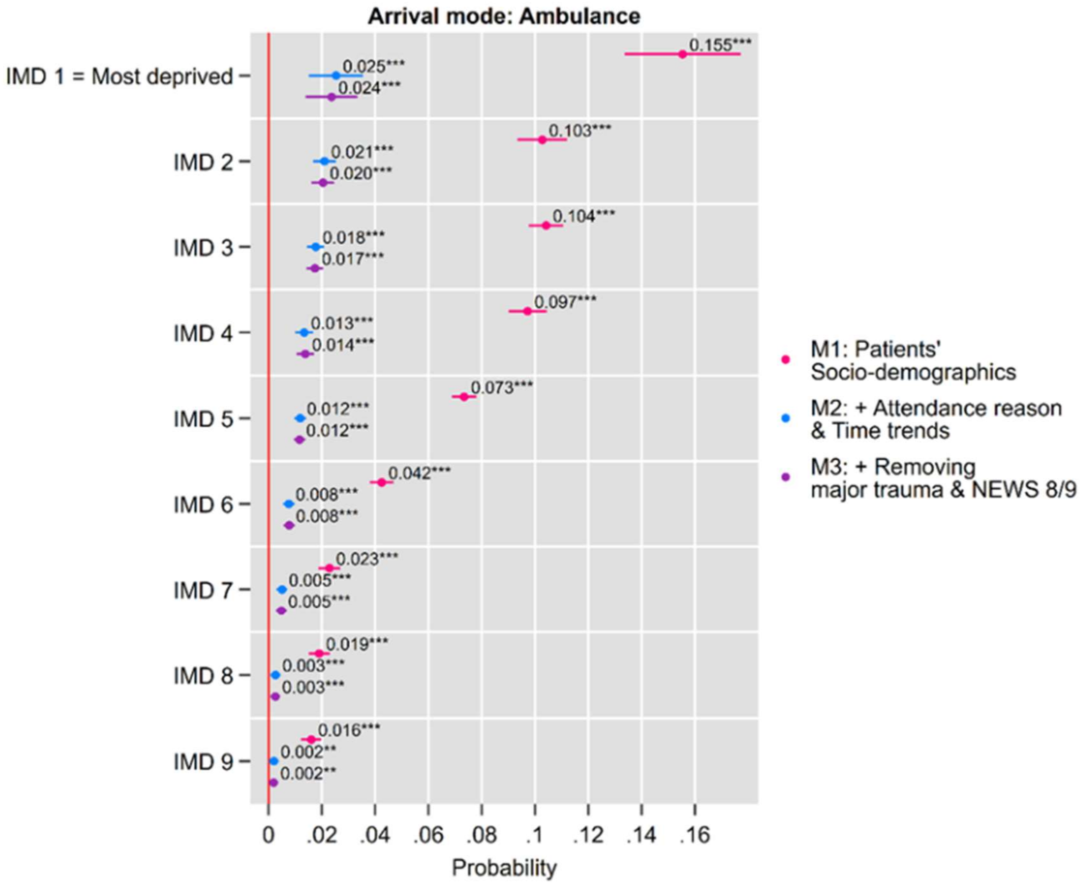

Supplement: Supplementary Figure 3 [file bmjopen-15-12-s003.pdf]
