# Supplementary material for: Socioeconomic inequality and access to emergency care: understanding the pathways to the emergency department in the UK
Source: BMJ Open. 2025 Dec 12;15(12):e108770. doi: 10.1136/bmjopen-2025-108770 (PMC12706212; doi:10.1136/bmjopen-2025-108770)

Figure A.4: Trends over time for Total time in ED, Stayed in ED for 4 hours or more, Admission probability, Unplanned returns

Panel A Total minutes in the Department

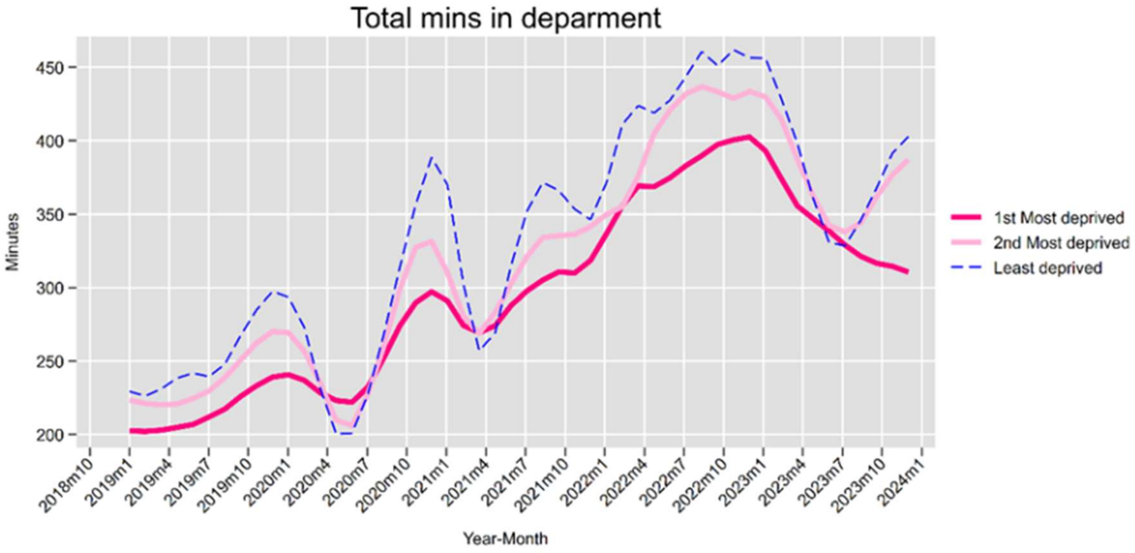

Panel B Stayed in ED for 4hr or more

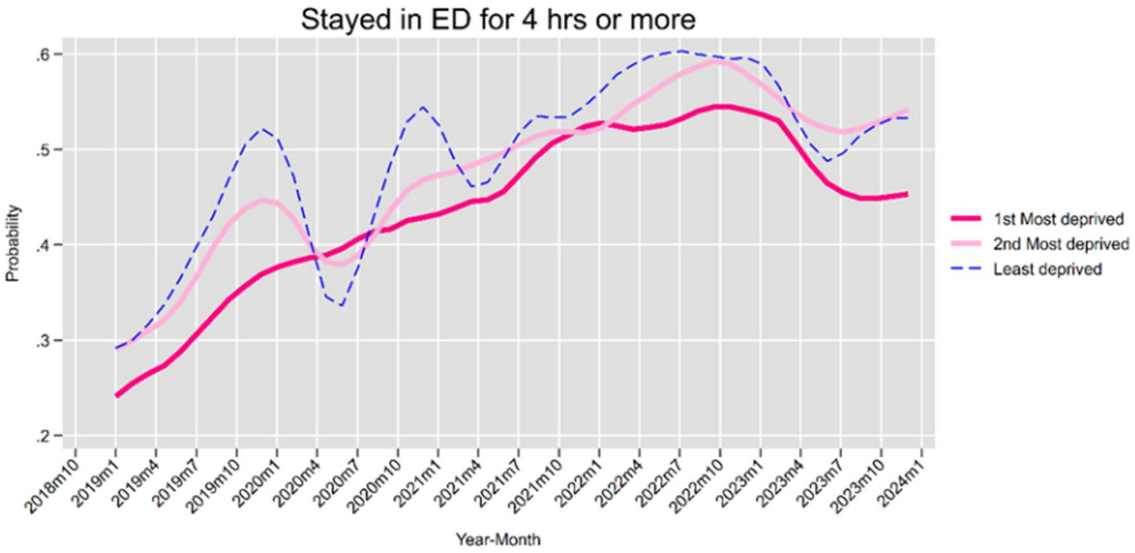

Panel C Hospital admission Probability

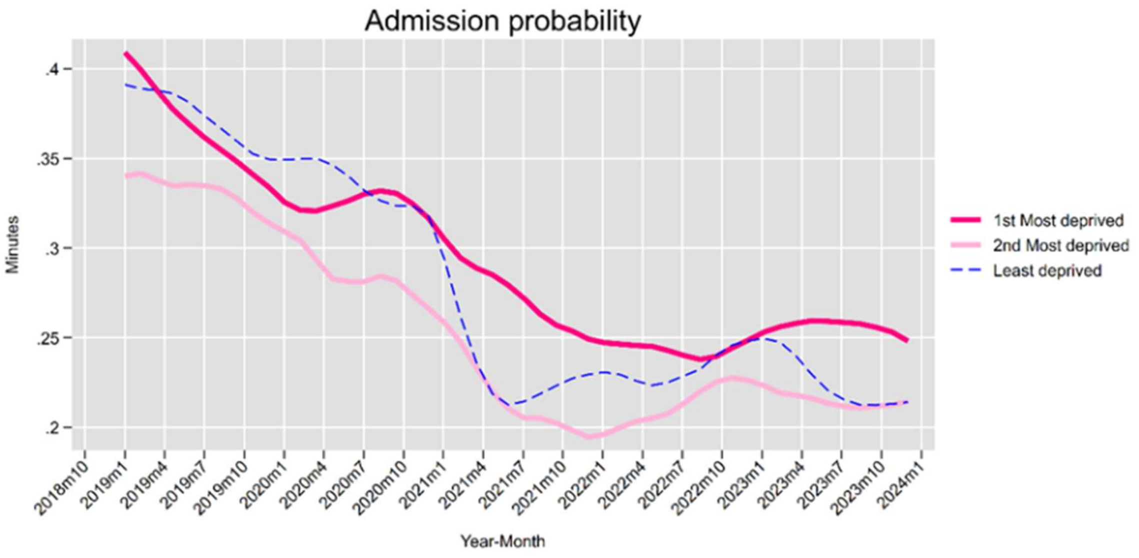

Panel D Unplanned returns

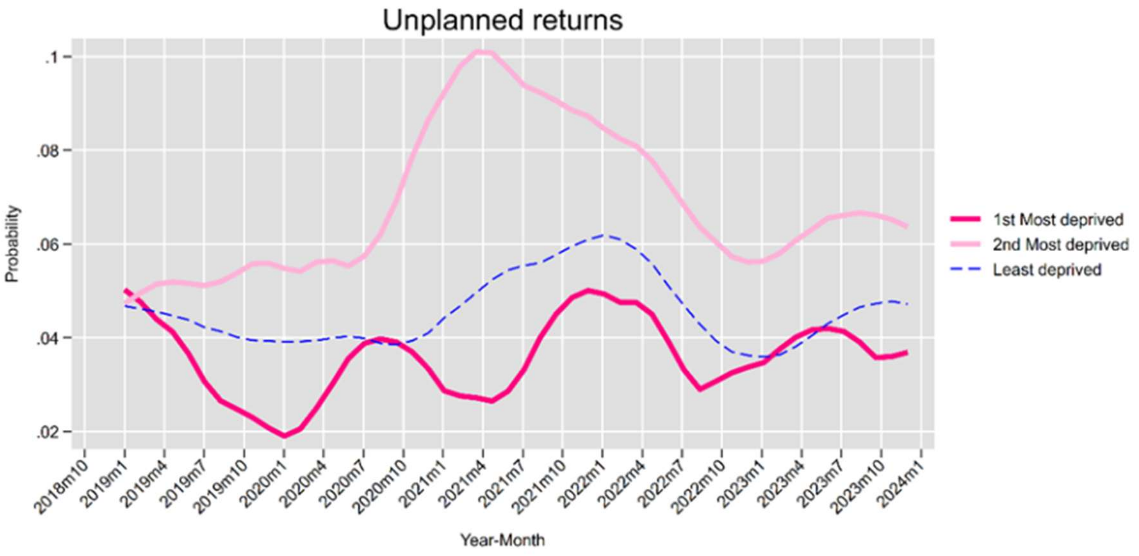

Supplement: Supplementary Figure 4 [file bmjopen-15-12-s004.pdf]
