# Supplementary material for: Socioeconomic inequality and access to emergency care: understanding the pathways to the emergency department in the UK
Source: BMJ Open. 2025 Dec 12;15(12):e108770. doi: 10.1136/bmjopen-2025-108770 (PMC12706212; doi:10.1136/bmjopen-2025-108770)

Figure A.5: Distribution of Admission probability by Total minutes in ED. This Figure shows the distribution of admission probability by total time spent in the ED, expressed in 5-minute intervals. Admission probability peaks at 240 minutes, which corresponds to the current NHS target for emergency department stays. This may indicate that clinicians experience pressure to make admission decisions as this threshold approaches.

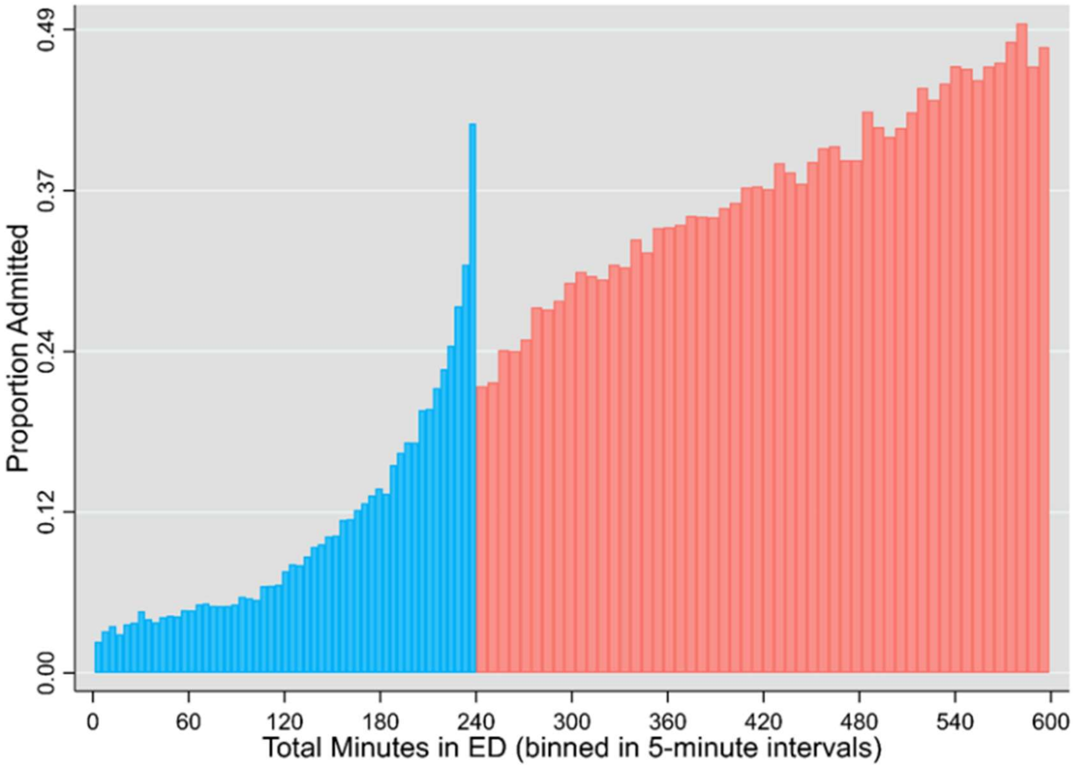

Supplement: Supplementary Figure 5 [file bmjopen-15-12-s005.pdf]
