# Supplementary material for: Socioeconomic inequality and access to emergency care: understanding the pathways to the emergency department in the UK
Source: BMJ Open. 2025 Dec 12;15(12):e108770. doi: 10.1136/bmjopen-2025-108770 (PMC12706212; doi:10.1136/bmjopen-2025-108770)
Supplement: Supplementary file 1 [file bmjopen-15-12-s010.pdf]

Supplement materials

Representativeness and Context of the Study Site

The data are drawn from Cambridge University Hospitals NHS Foundation Trust (CUH), located in the East of England. The local area ranks among the least deprived in England (Cambridge city IMD rank ≈ 7th decile nationally), though the hospital's catchment extends to surrounding Cambridgeshire districts, parts of Norfolk, Suffolk, and Essex, which include more socioeconomically diverse populations. According to NHS Digital's *Hospital Episode Statistics*, CUH's emergency attendance demographics are broadly comparable to other large regional teaching hospitals in terms of age and gender distribution, but the overall deprivation profile is moderately skewed toward less deprived deciles. Therefore, the results should be interpreted as representative of large, mixed-population hospitals rather than the national ED system as a whole.

Full list of Covariates

To ensure that the analysis accurately captures the relationship between socioeconomic deprivation (as measured by IMD) and emergency department outcomes, we control for a comprehensive set of covariates:

- **Sociodemographic Characteristics**
  - **Gender**: Binary variable coded as 1 for female and 0 for male.
  - **Age Group**: Categorical variable comprising the following groups: 16–24, 25–34, 35–44, 45–54, 55–64, 65–74, 75–84, and 85+ years.
  - **UK Residency Status**: Binary indicator coded as 1 for UK residents and 0 for non-residents.
- **Clinical Acuity**
  - **National Early Warning Score (NEWS)**: Categorical variable capturing physiological acuity on arrival, grouped as: No score, 0, 1, 2, 3, 4, 5, 6, 7, 8, and 9. Higher scores indicate greater acuity.
- **ED Care Pathway Variables**
  - **Referral Source**: Categorical variable indicating how the patient was referred to the ED: NHS 111, Ambulance, GP/Practice Nurse, Self-referral, Other medical professional, and Other non-medical professional.
  - **Arrival Mode**: Categorical variable detailing how the patient arrived: Emergency road ambulance, Non-emergency transport, Public/private transport, Air ambulance, Police/prison transport, or Unknown.
  - **Attendance Category**: Categorical variable classifying the presenting complaint or primary reason for visit, including: Major trauma, Injuries and trauma, Mental health issues, Pregnancy and gynaecological issues, Pain, Cardiac issues, Neurological issues, Infectious diseases, Substance abuse and poisoning, Gastrointestinal issues, Respiratory issues, Other, Don't know, and Direct referral to inpatient unit.

    - **ED Treatment Area**: Categorical variable indicating the clinical zone where the patient was directed: Majors (ambulant), Majors (trolley), Minors, or Resuscitation (resus).
- **Geographical Area of Residence**
    - Categorical variable indicating the patient's area of residence. The distribution in the study sample was as follows: Cambridge (28.6%), South Cambridgeshire (29.1%), East Cambridgeshire (12.0%), Huntingdonshire (2.7%), Other Cambridgeshire (1.1%), Suffolk (7.6%), Norfolk (0.8%), Essex and Hertfordshire (12.7%), Bedfordshire (1.1%), London & Greater London (1.1%), South East of England (0.2%), and the Rest of England (2.8%).
- **COVID-19 Status**
    - Categorical variable indicating isolation requirement or COVID-19 classification: COVID-19 confirmed, COVID-19 possible, No isolation required, Non-COVID, and Side room required (non-COVID reason).
- **Temporal and Operational Variables**
    - **Shift**: Binary variable indicating whether the attendance occurred during a night shift (22:00 to 08:00 = 1; otherwise = 0).
    - **Day of the Week**: Categorical variable for the day the patient attended ED.
    - **Month and Year of Arrival**: Categorical variables capturing temporal variation.
    - **ED Workload Proxies**:
        - **Total ED Arrivals**: Continuous variables representing the number of ED attendances per day, month, and year.
        - **Total Admissions**: Daily, monthly, and yearly admission counts used to proxy for hospital throughput and occupancy.
        - **3-Day Rolling Average of Arrivals**: Continuous variable used to reflect recent ED pressure and workload over time

Figure A.1: Development of analytical dataset

Figure A.2: Conceptual framework linking deprivation, referral source, and hospital outcomes.

The diagram illustrates the overall analytical framework linking area deprivation (IMD), referral source, and hospital outcomes. Model 1 focuses on the direct effect of IMD on referral source (blue path), showing how socioeconomic deprivation influences the way patients access the Emergency Department (e.g., GP/practice nurse, NHS 111, ambulance, or self-referral), after accounting for patient characteristics and temporal and hospital variables (dotted associations). Model 2 extends this framework to examine hospital outcomes. It incorporates both the direct effects of IMD (green path) and referral source (purple path) on outcomes such as admission probability, total ED time, four-hour breaches, and unplanned returns, as well as their interaction (IMD × Referral) (orange dashed path). The figure therefore represents the full causal structure, where Model 1 captures the pathway from deprivation to referral patterns, and Model 2 builds on it to evaluate how these factors jointly influence hospital outcomes.

Figure A.3: Probability of Arriving to the ED in an emergency road ambulance, AME, Additional results on Arrival mode: Emergency Road Ambulance

Figure A.4: Trends over time for Total time in ED, Stayed in ED for 4 hours or more, Admission probability, Unplanned returns

Panel A: Total minute in the Department

Panel B: Stayed in ED for 4hr or more

Panel C: Hospital admission Probability

Panel D: Unplanned returns

Figure A.5: Distribution of Admission probability by Total minutes in ED

This Figure shows the distribution of admission probability by total time spent in the ED, expressed in 5-minute intervals. Admission probability peaks at 240 minutes, which corresponds to the current NHS target for emergency department stays. This may indicate that clinicians experience pressure to make admission decisions as this threshold approaches.

Figure A.6: Absolute distribution of IMD deciles and Number of visits, 2019-2023

Figure A.7: Distribution of Catchment areas by Year, 2019-2023

Figure A.8: IMD Decile Distribution by Referral Source

Figure A.9: IMD Decile Distribution by Attendance Category

Table A.1: Summary Statistics

Variables	Mean	Count	SD	Min	Max
IMD Decile 1 (Most Deprived)	0.004	2,060	0.065	0	1
IMD Decile 2	0.024	11,354	0.152	0	1
IMD Decile 3	0.053	25,513	0.224	0	1
IMD Decile 4	0.040	19,480	0.197	0	1
IMD Decile 5	0.122	58,919	0.327	0	1
IMD Decile 6	0.114	54,811	0.317	0	1
IMD Decile 7	0.138	66,658	0.345	0	1
IMD Decile 8	0.157	75,630	0.363	0	1
IMD Decile 9	0.179	86,232	0.383	0	1
IMD Decile 10 (Least Deprived)	0.170	82,130	0.376	0	1
NHS 111 Referral	0.109	52,410	0.311	0	1
Ambulance Referral	0.271	130,710	0.444	0	1
GP or Practice Nurse Referral	0.132	63,912	0.339	0	1
Self-Referral	0.409	197,371	0.492	0	1
Other Medical Professional Referral	0.073	35,168	0.260	0	1
Other Non-Medical Professional Referral	0.007	3,216	0.081	0	1
Walk-in Arrival	0.693	334,608	0.461	0	1
Emergency Road Ambulance	0.288	139,144	0.453	0	1
Air Ambulance	0.001	529	0.033	0	1
Non-Emergency Ambulance	0.002	1,014	0.046	0	1
Police or Prison Transport	0.006	3,131	0.080	0	1
Unknown Arrival Mode	0.009	4,361	0.095	0	1
Female Patient	0.530	255,991	0.499	0	1
Age 16–24	0.147	70,771	0.354	0	1
Age 25–34	0.157	75,940	0.364	0	1
Age 35–44	0.129	62,192	0.335	0	1
Age 45–54	0.123	59,240	0.328	0	1
Age 55–64	0.119	57,690	0.324	0	1
Age 65–74	0.112	54,246	0.316	0	1
Age 75–84	0.122	59,014	0.328	0	1
Age 85+	0.091	43,694	0.287	0	1
Non-UK Resident	0.007	3,269	0.082	0	1
Cambridge	0.286	138,184	0.452	0	1
South Cambridgeshire	0.291	140,670	0.454	0	1
East Cambridgeshire	0.120	58,121	0.325	0	1
Huntingdonshire	0.027	13,237	0.163	0	1
Other Cambridgeshire	0.011	5,503	0.106	0	1
Suffolk	0.076	36,683	0.265	0	1
Norfolk	0.008	3,684	0.087	0	1
Essex and Hertfordshire	0.127	61,505	0.333	0	1

Bedfordshire	0.011	5,327	0.104	0	1
London and Greater London	0.011	5,453	0.106	0	1
South East England	0.002	995	0.045	0	1
Rest of England	0.028	13,425	0.164	0	1
NEWS2: No Score Recorded	0.256	123,713	0.437	0	1
NEWS2 Score 0	0.274	132,370	0.446	0	1
NEWS2 Score 1	0.214	103,516	0.410	0	1
NEWS2 Score 2	0.106	50,944	0.307	0	1
NEWS2 Score 3	0.061	29,374	0.239	0	1
NEWS2 Score 4	0.034	16,192	0.180	0	1
NEWS2 Score 5	0.020	9,810	0.141	0	1
NEWS2 Score 6	0.014	6,750	0.117	0	1
NEWS2 Score 7	0.009	4,530	0.096	0	1
NEWS2 Score 8	0.007	3,241	0.082	0	1
NEWS2 Score 9	0.005	2,347	0.070	0	1
Major Trauma	0.004	2,171	0.067	0	1
Injuries and Trauma	0.278	133,980	0.448	0	1
Mental Health Issues	0.030	14,376	0.170	0	1
Pregnancy and Gynaecological Issues	0.013	6,068	0.111	0	1
Pain	0.080	38,760	0.272	0	1
Cardiac Issues	0.106	51,162	0.308	0	1
Neurological Issues	0.164	79,142	0.370	0	1
Infectious Diseases	0.055	26,536	0.228	0	1
Substance Abuse or Poisoning	0.007	3,587	0.086	0	1
Gastrointestinal Issues	0.118	56,836	0.322	0	1
Respiratory Issues	0.062	30,032	0.242	0	1
Other Presentations	0.046	21,995	0.209	0	1
Unknown Presentation	0.008	3,836	0.089	0	1
Direct Referral to Inpatient Unit	0.030	14,306	0.170	0	1
Majors (Ambulant)	0.455	219,627	0.498	0	1
Majors (Trolley)	0.228	110,075	0.420	0	1
Minors	0.287	138,602	0.452	0	1
Paediatric Area	0.001	284	0.024	0	1
Resuscitation Area	0.016	7,823	0.126	0	1
Area Unknown / Missing	0.013	6,376	0.114	0	1
COVID-19 Confirmed	0.014	68,09	0.118	0	1
COVID-19 Possible	0.036	17,351	0.186	0	1
No Isolation Requirement	0.644	310,807	0.479	0	1
Non-COVID Case	0.282	136,201	0.450	0	1
Side Room Required (Other Reason)	0.024	11,619	0.153	0	1
Night Shift Attendance	0.174	83,853	0.379	0	1
Sunday	0.136	65,693	0.343	0	1
Monday	0.156	75,294	0.363	0	1
Tuesday	0.146	70,392	0.353	0	1
Wednesday	0.142	68,763	0.349	0	1
Thursday	0.143	69,023	0.350	0	1

Friday	0.141	68,062	0.348	0	1
Saturday	0.136	65,560	0.343	0	1
January	0.081	38,975	0.272	0	1
February	0.078	37,875	0.269	0	1
March	0.084	40,363	0.277	0	1
April	0.079	37,914	0.269	0	1
May	0.087	42,215	0.282	0	1
June	0.084	40,322	0.277	0	1
July	0.081	38,899	0.272	0	1
August	0.090	43,262	0.286	0	1
September	0.088	42,298	0.283	0	1
October	0.092	44,267	0.289	0	1
November	0.074	35,643	0.261	0	1
December	0.084	40,754	0.278	0	1
Arrival Year: 2019	0.209	100,986	0.407	0	1
Arrival Year: 2020	0.174	83,849	0.379	0	1
Arrival Year: 2021	0.200	96,389	0.400	0	1
Arrival Year: 2022	0.216	104,237	0.411	0	1
Arrival Year: 2023	0.202	97,326	0.401	0	1
Daily ED Arrivals (Count)	359.6	-	62.3	2	561
Daily ED Admissions (Count)	83.3	-	20.2	0	152
3-Day Rolling Average of ED Arrivals	357.6	-	57.5	3	525
Total Observations	482,787				

Table A.2: Average Marginal Effects (AME) of IMD Deciles on the Probability of Being Referred via Different Referral Sources

IMD Decile (vs. IMD 10)	NHS 111	Ambulance	GP/Practice	Self-referral	Other medical	Other non-medical
IMD 1	-0.028***	0.081***	-0.046***	-0.033***	-0.002	0.013***
(most deprived)	(0.006)	(0.009)	(0.008)	(0.010)	(0.005)	(0.002)
IMD 2	-0.024***	0.044***	-0.010***	-0.014***	0.002	0.006***
	(0.003)	(0.004)	(0.003)	(0.004)	(0.003)	(0.001)
IMD 3	-0.010***	0.056***	-0.012***	-0.026***	-0.009***	0.003***
	(0.002)	(0.003)	(0.003)	(0.003)	(0.002)	(0.001)
IMD 4	-0.011***	0.049***	-0.012***	-0.024***	-0.006***	0.005***
	(0.003)	(0.003)	(0.003)	(0.004)	(0.002)	(0.001)
IMD 5	-0.007***	0.036***	-0.012***	-0.014***	-0.006***	0.003***
	(0.002)	(0.002)	(0.002)	(0.002)	(0.001)	(0.000)
IMD 6	-0.007***	0.018***	-0.007***	-0.008***	-0.002	0.004***
	(0.002)	(0.002)	(0.002)	(0.003)	(0.001)	(0.000)
IMD 7	-0.008***	0.008***	-0.006***	0.004*	-0.001	0.001***
	(0.002)	(0.002)	(0.002)	(0.002)	(0.001)	(0.000)
IMD 8	-0.001	0.013***	-0.003*	-0.005**	-0.006***	0.000
	(0.002)	(0.002)	(0.002)	(0.002)	(0.001)	(0.000)
IMD 9	0.001	0.010***	-0.001	-0.009***	-0.003**	0.000
	(0.002)	(0.002)	(0.002)	(0.002)	(0.001)	(0.000)

Sociodemographic controls	Yes	Yes	Yes	Yes	Yes	Yes
Clinical controls	Yes	Yes	Yes	Yes	Yes	Yes
Temporal controls	Yes	Yes	Yes	Yes	Yes	Yes
System pressure controls	Yes	Yes	Yes	Yes	Yes	Yes
Observations	482,787	482,787	482,787	482,787	482,787	482,787

Notes: Average marginal effects (AMEs) from logistic regression models. Coefficients are in probability units; standard errors in parentheses clustered at patient level. IMD 10 (least deprived) is the reference category. *** p < 0.01, ** p < 0.05, * p < 0.10. Sociodemographic controls include patient sex, age group, non-UK residency status, and area of residence (Cambridge, South Cambridgeshire, East Cambridgeshire, etc.). Clinical controls include the NEWS2 score, attendance category (e.g. injuries, cardiac, respiratory, mental health), hospital area (majors, minors, resuscitation, paediatric), and COVID-19 isolation status. Temporal controls include day of week, month, year of attendance, and whether the attendance occurred during a night shift. System pressure controls include the total number of daily emergency department arrivals, daily emergency admissions, and the three-day rolling average of arrivals (as proxies for crowding and workload).

Table A.3: IMD-Referral Source Interactions, Hospital admission probability, Average Marginal Effects

IMD10#Referral	AME	Std. Err.	95% CI Lower	95% CI Upper
1#111	0.246	0.008	0.23	0.261
1#Ambulance	0.274	0.008	0.257	0.29
1#GP/Practice Nurse	0.291	0.009	0.274	0.308
1#Self-referral	0.269	0.008	0.253	0.285
1#Other med prof.	0.348	0.009	0.331	0.366
1#Other non-med prof.	0.243	0.013	0.217	0.268
2#111	0.236	0.004	0.229	0.244
2#Ambulance	0.264	0.004	0.256	0.271
2#GP/Practice Nurse	0.281	0.004	0.273	0.288
2#Self-referral	0.259	0.004	0.252	0.266
2#Other med prof.	0.338	0.004	0.329	0.346
2#Other non-med prof.	0.233	0.011	0.212	0.255
3#111	0.235	0.003	0.229	0.24
3#Ambulance	0.262	0.003	0.257	0.267
3#GP/Practice Nurse	0.279	0.003	0.274	0.285
3#Self-referral	0.257	0.003	0.252	0.263
3#Other med prof.	0.336	0.003	0.329	0.342
3#Other non-med prof.	0.232	0.011	0.211	0.253
4#111	0.238	0.003	0.232	0.244
4#Ambulance	0.265	0.003	0.26	0.271
4#GP/Practice Nurse	0.282	0.003	0.276	0.288
4#Self-referral	0.261	0.003	0.255	0.266
4#Other med prof.	0.339	0.003	0.332	0.346
4#Other non-med prof.	0.235	0.011	0.214	0.256
5#111	0.241	0.002	0.236	0.246

5#Ambulance	0.269	0.002	0.265	0.273
5#GP/Practice Nurse	0.286	0.002	0.282	0.29
5#Self-referral	0.264	0.002	0.26	0.268
5#Other med prof.	0.343	0.003	0.338	0.348
5#Other non-med prof.	0.238	0.011	0.217	0.259
6#111	0.243	0.002	0.238	0.247
6#Ambulance	0.27	0.002	0.266	0.275
6#GP/Practice Nurse	0.288	0.002	0.283	0.292
6#Self-referral	0.266	0.002	0.262	0.269
6#Other med prof.	0.345	0.003	0.34	0.35
6#Other non-med prof.	0.24	0.011	0.219	0.261
7#111	0.24	0.002	0.236	0.244
7#Ambulance	0.268	0.002	0.264	0.272
7#GP/Practice Nurse	0.285	0.002	0.281	0.289
7#Self-referral	0.263	0.002	0.259	0.267
7#Other med prof.	0.342	0.002	0.337	0.347
7#Other non-med prof.	0.237	0.011	0.217	0.258
8#111	0.238	0.002	0.234	0.242
8#Ambulance	0.266	0.002	0.262	0.27
8#GP/Practice Nurse	0.283	0.002	0.279	0.287
8#Self-referral	0.261	0.002	0.258	0.265
8#Other med prof.	0.34	0.002	0.335	0.345
8#Other non-med prof.	0.235	0.011	0.215	0.256
9#111	0.238	0.002	0.234	0.242
9#Ambulance	0.266	0.002	0.262	0.269
9#GP/Practice Nurse	0.283	0.002	0.279	0.287
9#Self-referral	0.261	0.002	0.257	0.264
9#Other med prof.	0.34	0.002	0.335	0.344
9#Other non-med prof.	0.235	0.01	0.215	0.256
10#111	0.238	0.002	0.234	0.242
10#Ambulance	0.266	0.002	0.262	0.269
10#GP/Practice Nurse	0.283	0.002	0.279	0.287
10#Self-referral	0.261	0.002	0.257	0.264
10#Other med prof.	0.34	0.002	0.335	0.344
10#Other non-med prof.	0.235	0.01	0.215	0.256

Table A.4: IMD-Referral Source Interactions, Total time in ED, Average Marginal Effects

IMD10#Referral	AME	Std. Err.	95% CI Lower	95% CI Upper
1#111	311.3	5.1	301.2	321.3
1#Ambulance	351.4	5.8	340	362.8
1#GP/Practice Nurse	333.3	5.5	322.5	344.1
1#Self-referral	320.3	5.2	310	330.6
1#Other med prof.	321.9	5.4	311.4	332.4

1#Other non-med prof.	328.7	8.4	312.1	345.3
2#111	315.1	2.4	310.3	319.8
2#Ambulance	355.7	2.7	350.3	361
2#GP/Practice Nurse	337.4	2.6	332.4	342.4
2#Self-referral	324.2	2.4	319.6	328.8
2#Other med prof.	325.8	2.6	320.7	330.9
2#Other non-med prof.	332.7	7.1	318.9	346.5
3#111	315.6	1.8	312	319.2
3#Ambulance	356.3	2.1	352.3	360.3
3#GP/Practice Nurse	338	1.9	334.2	341.8
3#Self-referral	324.8	1.7	321.4	328.2
3#Other med prof.	326.4	2	322.4	330.4
3#Other non-med prof.	333.3	6.9	319.8	346.8
4#111	310.6	2	306.8	314.5
4#Ambulance	350.7	2.2	346.3	355
4#GP/Practice Nurse	332.6	2.1	328.6	336.7
4#Self-referral	319.6	1.9	316	323.3
4#Other med prof.	321.2	2.2	317	325.4
4#Other non-med prof.	328	6.8	314.7	341.4
5#111	311.2	1.4	308.4	314
5#Ambulance	351.3	1.6	348.2	354.5
5#GP/Practice Nurse	333.3	1.5	330.4	336.2
5#Self-referral	320.3	1.2	317.8	322.7
5#Other med prof.	321.8	1.6	318.6	325.1
5#Other non-med prof.	328.7	6.7	315.6	341.7
6#111	310.8	1.4	308	313.6
6#Ambulance	350.9	1.6	347.7	354
6#GP/Practice Nurse	332.8	1.5	329.9	335.8
6#Self-referral	319.8	1.3	317.3	322.3
6#Other med prof.	321.4	1.7	318.2	324.7
6#Other non-med prof.	328.2	6.7	315.1	341.3
7#111	306.8	1.4	304.2	309.5
7#Ambulance	346.4	1.5	343.4	349.4
7#GP/Practice Nurse	328.6	1.4	325.8	331.4
7#Self-referral	315.8	1.2	313.5	318.1
7#Other med prof.	317.3	1.6	314.2	320.4
7#Other non-med prof.	324	6.6	311.2	336.9
8#111	309.9	1.3	307.3	312.5
8#Ambulance	349.8	1.5	346.9	352.8
8#GP/Practice Nurse	331.9	1.4	329.2	334.6
8#Self-referral	318.9	1.2	316.7	321.2
8#Other med prof.	320.5	1.6	317.4	323.6
8#Other non-med prof.	327.3	6.6	314.3	340.2
9#111	307.7	1.3	305.1	310.3
9#Ambulance	347.3	1.5	344.5	350.2
9#GP/Practice Nurse	329.5	1.4	326.8	332.1

9#Self-referral	316.6	1.1	314.4	318.8
9#Other med prof.	318.2	1.5	315.2	321.2
9#Other non-med prof.	324.9	6.6	312	337.8
10#111	307.3	1.3	304.7	309.9
10#Ambulance	346.9	1.5	344	349.8
10#GP/Practice Nurse	329.1	1.4	326.4	331.8
10#Self-referral	316.2	1.1	314	318.5
10#Other med prof.	317.8	1.6	314.7	320.8
10#Other non-med prof.	324.5	6.6	311.6	337.4

Notes: Average marginal effects (AMEs) from logistic regression models. Coefficients are in probability units; standard errors in parentheses clustered at patient level. IMD 10 (least deprived) is the reference category. *** p < 0.01, ** p < 0.05, * p < 0.10. Sociodemographic controls include patient sex, age group, non-UK residency status, and area of residence (Cambridge, South Cambridgeshire, East Cambridgeshire, etc.). Clinical controls include the NEWS2 score, attendance category (e.g. injuries, cardiac, respiratory, mental health), hospital area (majors, minors, resuscitation, paediatric), and COVID-19 isolation status. Temporal controls include day of week, month, year of attendance, and whether the attendance occurred during a night shift. System pressure controls include the total number of daily emergency department arrivals, daily emergency admissions, and the three-day rolling average of arrivals (as proxies for crowding and workload).

Table A.5: IMD-Referral Source Interactions, Probability of staying in ED 4 hours or more, Average Marginal Effects

**IMD10#Referral**	**AME**	**Std. Err.**	**95% CI Lower**	**95% CI Upper**
1#111	0.48	0.01	0.461	0.499
1#Ambulance	0.512	0.01	0.493	0.531
1#GP/Practice Nurse	0.499	0.01	0.48	0.518
1#Self-referral	0.476	0.01	0.457	0.495
1#Other med prof.	0.486	0.01	0.467	0.505
1#Other non-med prof.	0.484	0.015	0.454	0.513
2#111	0.491	0.004	0.482	0.499
2#Ambulance	0.523	0.005	0.514	0.531
2#GP/Practice Nurse	0.51	0.004	0.501	0.518
2#Self-referral	0.487	0.004	0.478	0.495
2#Other med prof.	0.497	0.005	0.488	0.506
2#Other non-med prof.	0.494	0.012	0.47	0.519
3#111	0.492	0.003	0.485	0.499
3#Ambulance	0.524	0.003	0.517	0.531
3#GP/Practice Nurse	0.511	0.003	0.505	0.518
3#Self-referral	0.488	0.003	0.482	0.494
3#Other med prof.	0.498	0.004	0.491	0.505
3#Other non-med prof.	0.496	0.012	0.472	0.519
4#111	0.487	0.004	0.48	0.494
4#Ambulance	0.519	0.004	0.512	0.527

4#GP/Practice Nurse	0.507	0.004	0.5	0.513
4#Self-referral	0.483	0.003	0.477	0.49
4#Other med prof.	0.493	0.004	0.486	0.501
4#Other non-med prof.	0.491	0.012	0.467	0.515
5#111	0.485	0.003	0.48	0.49
5#Ambulance	0.517	0.003	0.511	0.522
5#GP/Practice Nurse	0.504	0.002	0.499	0.509
5#Self-referral	0.481	0.002	0.477	0.485
5#Other med prof.	0.491	0.003	0.485	0.496
5#Other non-med prof.	0.488	0.012	0.465	0.512
6#111	0.488	0.003	0.482	0.493
6#Ambulance	0.52	0.003	0.514	0.525
6#GP/Practice Nurse	0.507	0.002	0.502	0.512
6#Self-referral	0.484	0.002	0.479	0.488
6#Other med prof.	0.494	0.003	0.488	0.499
6#Other non-med prof.	0.491	0.012	0.468	0.515
7#111	0.481	0.003	0.476	0.486
7#Ambulance	0.513	0.003	0.508	0.519
7#GP/Practice Nurse	0.501	0.002	0.496	0.505
7#Self-referral	0.477	0.002	0.473	0.481
7#Other med prof.	0.487	0.003	0.482	0.493
7#Other non-med prof.	0.485	0.012	0.461	0.509
8#111	0.482	0.002	0.478	0.487
8#Ambulance	0.514	0.003	0.509	0.52
8#GP/Practice Nurse	0.502	0.002	0.497	0.506
8#Self-referral	0.478	0.002	0.475	0.482
8#Other med prof.	0.488	0.003	0.483	0.494
8#Other non-med prof.	0.486	0.012	0.463	0.51
9#111	0.482	0.002	0.477	0.486
9#Ambulance	0.514	0.003	0.509	0.519
9#GP/Practice Nurse	0.501	0.002	0.497	0.505
9#Self-referral	0.478	0.002	0.474	0.481
9#Other med prof.	0.488	0.003	0.482	0.493
9#Other non-med prof.	0.485	0.012	0.462	0.509
10#111	0.478	0.002	0.473	0.483
10#Ambulance	0.51	0.003	0.505	0.515
10#GP/Practice Nurse	0.497	0.002	0.493	0.502
10#Self-referral	0.474	0.002	0.47	0.478
10#Other med prof.	0.484	0.003	0.478	0.489
10#Other non-med prof.	0.481	0.012	0.458	0.505

Notes: Average marginal effects (AMEs) from GLM regression models. Coefficients are in minute units; standard errors in parentheses clustered at patient level. IMD 10 (least deprived) is the reference category. *** p < 0.01, ** p < 0.05, * p < 0.10. Sociodemographic controls include patient sex, age group, non-UK residency status, and area of residence (Cambridge, South Cambridgeshire, East Cambridgeshire, etc.). Clinical controls include the NEWS2 score, attendance category (e.g. injuries, cardiac, respiratory, mental health), hospital area (majors, minors, resuscitation, paediatric), and

COVID-19 isolation status. Temporal controls include day of week, month, year of attendance, and whether the attendance occurred during a night shift. System pressure controls include the total number of daily emergency department arrivals, daily emergency admissions, and the three-day rolling average of arrivals (as proxies for crowding and workload).

Table A.6: IMD-Referral Source Interactions, Unplanned returns to ED, Average Marginal Effects

IMD10#Referral	AME	Std. Err.	95% CI Lower	95% CI Upper
1#111	0.029	0.003	0.022	0.036
1#Ambulance	0.047	0.006	0.036	0.058
1#GP/Practice Nurse	0.032	0.004	0.025	0.04
1#Self-referral	0.071	0.008	0.055	0.087
1#Other med prof.	0.054	0.006	0.042	0.066
1#Other non-med prof.	0.054	0.009	0.037	0.071
2#111	0.037	0.002	0.033	0.04
2#Ambulance	0.06	0.003	0.055	0.065
2#GP/Practice Nurse	0.041	0.002	0.037	0.044
2#Self-referral	0.089	0.003	0.083	0.096
2#Other med prof.	0.068	0.003	0.063	0.074
2#Other non-med prof.	0.068	0.008	0.053	0.084
3#111	0.027	0.001	0.025	0.03
3#Ambulance	0.045	0.002	0.042	0.048
3#GP/Practice Nurse	0.031	0.001	0.028	0.033
3#Self-referral	0.068	0.002	0.064	0.072
3#Other med prof.	0.051	0.002	0.048	0.055
3#Other non-med prof.	0.052	0.006	0.04	0.064
4#111	0.032	0.001	0.029	0.034
4#Ambulance	0.052	0.002	0.047	0.056
4#GP/Practice Nurse	0.035	0.001	0.032	0.038
4#Self-referral	0.077	0.003	0.072	0.082
4#Other med prof.	0.059	0.002	0.054	0.063
4#Other non-med prof.	0.059	0.007	0.045	0.073
5#111	0.026	0.001	0.024	0.028
5#Ambulance	0.043	0.001	0.04	0.046
5#GP/Practice Nurse	0.029	0.001	0.027	0.031
5#Self-referral	0.065	0.001	0.062	0.067
5#Other med prof.	0.049	0.001	0.046	0.052
5#Other non-med prof.	0.049	0.006	0.038	0.06
6#111	0.026	0.001	0.024	0.028
6#Ambulance	0.043	0.001	0.04	0.045
6#GP/Practice Nurse	0.029	0.001	0.027	0.031
6#Self-referral	0.064	0.001	0.062	0.067
6#Other med prof.	0.049	0.002	0.046	0.052
6#Other non-med prof.	0.049	0.006	0.038	0.06

7#111	0.027	0.001	0.025	0.028
7#Ambulance	0.044	0.001	0.041	0.046
7#GP/Practice Nurse	0.03	0.001	0.028	0.031
7#Self-referral	0.066	0.001	0.063	0.068
7#Other med prof.	0.05	0.001	0.047	0.053
7#Other non-med prof.	0.05	0.006	0.039	0.061
8#111	0.025	0.001	0.024	0.027
8#Ambulance	0.042	0.001	0.039	0.044
8#GP/Practice Nurse	0.028	0.001	0.027	0.03
8#Self-referral	0.063	0.001	0.061	0.066
8#Other med prof.	0.048	0.001	0.045	0.051
8#Other non-med prof.	0.048	0.006	0.037	0.059
9#111	0.026	0.001	0.024	0.027
9#Ambulance	0.042	0.001	0.04	0.045
9#GP/Practice Nurse	0.029	0.001	0.027	0.03
9#Self-referral	0.064	0.001	0.061	0.066
9#Other med prof.	0.048	0.001	0.046	0.051
9#Other non-med prof.	0.048	0.006	0.037	0.059
10#111	0.025	0.001	0.024	0.027
10#Ambulance	0.041	0.001	0.039	0.044
10#GP/Practice Nurse	0.028	0.001	0.027	0.03
10#Self-referral	0.063	0.001	0.06	0.065
10#Other med prof.	0.048	0.001	0.045	0.05
10#Other non-med prof.	0.048	0.006	0.037	0.059

Notes: Average marginal effects (AMEs) from logistic regression models. Coefficients are in probability units; standard errors in parentheses clustered at patient level. IMD 10 (least deprived) is the reference category. *** $p < 0.01$, ** $p < 0.05$, * $p < 0.10$. Sociodemographic controls include patient sex, age group, non-UK residency status, and area of residence (Cambridge, South Cambridgeshire, East Cambridgeshire, etc.). Clinical controls include the NEWS2 score, attendance category (e.g. injuries, cardiac, respiratory, mental health), hospital area (majors, minors, resuscitation, paediatric), and COVID-19 isolation status. Temporal controls include day of week, month, year of attendance, and whether the attendance occurred during a night shift. System pressure controls include the total number of daily emergency department arrivals, daily emergency admissions, and the three-day rolling average of arrivals (as proxies for crowding and workload).
