# Supplementary material for: Socioeconomic inequality and access to emergency care: understanding the pathways to the emergency department in the UK
Source: BMJ Open. 2025 Dec 12;15(12):e108770. doi: 10.1136/bmjopen-2025-108770 (PMC12706212; doi:10.1136/bmjopen-2025-108770)
Supplement: Supplementary file 11 [file bmjopen-2025-108770.reviewer_comments.pdf]

PEER REVIEW HISTORY

BMJ Open publishes all reviews undertaken for accepted manuscripts. Reviewers are asked to complete a checklist review form and are provided with free text boxes to elaborate on their assessment. These free text comments are reproduced below.

ARTICLE DETAILS

Title (Provisional)

Socioeconomic Inequality and Access to Emergency Care: Understanding the Pathways to the Emergency Department

Authors

Madia, Joan; Boyle, Adrian A; Ray, James; Novak, Alex; Pope, Catherine J; Wheeler, Bella; Petrou, Stavros; Wittenberg, Raphael; Nicodemo, Catia

---

VERSION 1 - REVIEW

**Reviewer**	**1**
**Name**	**Gao , Ni**
**Affiliation**	**University of York**
**Date**	**19-Sep-2025**
**COI**	**None**

Thank you for sharing this manuscript with me. This manuscript addresses an important question using rich data. The manuscript needs improve clarity in some sections and more descriptive tables or figures are needed to help readers understand the whole story. My main comment is you need to explain why you think referral pathways affect downstream outcomes. From economic perspective, perhaps explain from supply and demand of healthcare. From medical perspective, doctors treat patients given the severity of diseases rather than referral routes. Some referral routes may imply less severe diseases so doctors may let them wait longer for the treatment. All in all, you need a theory or framework to support your research questions.

The following are my detailed comments.

Title: Perhaps think of a title that catches eyes? The current title has two key elements, socioeconomic inequalities and referral pathways, which make me wonder which is the key information that you want to convey?

Introduction: You have mentioned NHS in the first three paragraphs. Given the journal opens to the international readers, perhaps use one or two sentences to explain NHS, just as how you have explained US healthcare system.

In the 4th paragraph, you combine aims and contributions together. From logic flow perspective, aims should be in the first place so that it connects well with previous paragraph. Also for the 3rd paragraph, you may consider delete ‘however’ (it is better to

avoid using 'despite' or 'however' as the first word of the first sentence. This is just suggestion; you can stick with your writing style).

Still in the 4th paragraph, you address two questions. It would be good mention briefly the framework or the theories that you rely on to address those questions. For example, why different referral sources affect downstream outcomes? If a patient has heart attack and is admitted to ED. Doctors will treat them with priority regardless the referral route they use.

Also, some referral routes may be suitable for less emergent diseases. That is, referral sources may be affected by types of diseases, and the downstream outcomes just reflect the differences in diseases not the types of referral routes. Just justify.

The last paragraph seems connects well with two questions in the 4th paragraph. Pls consider improving logic flow and coherence.

Methods: Perhaps in Appendix, you may explain how representative of your sample is. As this is from one hospital, where does it locate (in more or less deprived areas)? Also, the data is from 2019 to 2024, may need a few words to explain whether the pandemic affects your analysis.

In the 2nd paragraph in the Method section, pls consider improving clarity. You use referral sources first as outcomes to examine whether they vary by IMD and then as an explanatory variable to examine whether downstream outcomes vary by referral sources and by IMD. Here, do you mean ten IMD dummies interacted with six referral dummies? All in one equation? It would be easier to explain in equation. The last sentence in this paragraph is redundant.

For outcomes, as there are international readers, please write a few words that 4 hours is a national target in UK. Also clearly indicate which outcomes are continuous and which are binary, as you mention to use logic and GLM in the later paragraphs.

Results: I am confused with 1st paragraph, there is no table or figure to show such disparities before reaching ED. If there are main findings, it comes so directly before readers can digest what your data look like. Or your explanation is based on Figure 1? Figure 1 itself is a bit confusing. I don't find arrival mode (lower panel).

Is there a typo that Figure 2 should be Figure 1? Also, I am wondering whether to present another figure from alternative angle, the distribution of IMD for each referral source? Similarly, for Figure 2 showing the attendance reasons, is it possible to show within each IMD, the distribution of different reasons. This can help readers to understand different attendance reasons for different IMD. Additionally, the crosstabulation between attendance reasons and referral routes. Figure 2 itself needs more clarity such as what x-axis is?

Before showing all those figures, it would be good to just show number of ED visits in each IMD using ranked bar chart. Some studies using data from a single hospital shows much higher visits from IMD10, so worth checking this first.

On page 7, the second paragraph, I get lost again as you don't mention which figure I need to read to help me understand your description. The 3rd paragraph should be in method section. I am a bit confused with the model you use. Am I right that you include IMD dummies with each referral source as an outcome, and IMD10 is the reference group? If so, can you improve clarity?

Figure 3: pls provide note below the figure (add '0' on x-scale) to help explain the figure. Pls use different legend shapes, considering white and black printing. Also explain why only examine three referral sources rather than six. Explain how you calculate probability (difference between each IMD with IMD10?) Have you detailed the analysis for each attendance reason (the first three common reasons)? Or adding interaction term between IMD and most three common attendance reasons?

Figure 4: The title should be hospital admission? I am wondering can you provide a table showing three models: i) IMD as key variable; ii) IMD and referral sources as key variables; and iii) IMD+referral sources+interaction terms.

On page 8, Figure 4 should be Figure 5, and the same typo in the following paragraphs. For Figure 5, it is total time, so should the title and y-axis be different?

For Figure 4-7: I am wondering whether there is alternative way to show the difference across IMD. Is it possible to use AME as a benchmark, and show difference between AME from each IMD with overall AME?

**Reviewer**	**2**
**Name**	**Dack, Kyle**
**Affiliation**	**University of Bristol, MRC Integrative Epidemiology Unit**
**Date**	**01-Oct-2025**
**COI**	**None**

Statistical review summary:

The study design, analysis plan and interpretation are all reasonable and I do not see major risks of bias. However, the organization of the paper is somewhat muddled, and this undermines the clarity of the paper. Additionally, the statistical analysis is described very briefly and this makes it challenging to follow the results section. Many of the comments below overlap with the STROBE checklist, which I recommend the authors cross-reference, including the elaboration document which has helpful examples.

Abstract:

1. The abstract is for the most part clear, and contains all essential information for readers to get an understanding of the study. However, regarding the statistical reporting I recommend aligning with STROBE guidance and including an uncertainty measure for each comparison (e.g. p-value or 95% CI).

Introduction:

2. “A study using national data found a 50% higher ED attendance rate in the most deprived areas compared to the least deprived” is it possible to also report the absolute percentages for the two groups?

Methods:

3. I suggest applying headings to make this easier to follow, such study setting, participants, data source/linkage, outcomes, variables, statistical analyses. There is not currently a publication checklist applied and I suggest STROBE.

4. Participants: How were multiple visits by the same participants handled? Was any deduplication applied such as restricting to the first visit, or were multiple visits permitted? I imagine it is the latter as the unit of analysis is always described as “visits” but it would be good to directly confirm this in the methods.

5. The study engages with an interesting question regarding how deprivation and referral pathway interact in ED attendances. This involved testing whether referral pathway varied by deprivation, and evaluating the association of both with various ED-related outcomes. Something which would be very useful is if it was possible for the authors to show the assumed relationship between these variables and the other covariates, i.e. using a DAG, to allow the potential causal relationships to be visualized. This would also help clarify the rationale for the various covariates which are described in paragraph 4 – which are assumed to be confounders, and which are competing exposures adjusted for to reduce uncertainty?

6. Statistical analysis: this section lacks details which impact the transparency and clarity of the analysis, and it would be helpful to explain (a) how each model aligns with the aims or how the result is intended to be interpreted, (b) the full specification of each model, i.e. the outcome and covariates and (c) more information about the GLM – which family specifications were considered, and how was non-normality determined and for which outcomes.

Results

7. There are certain elements which seem more suitable to the discussion, as the focus is on interpreting findings rather than presenting them. In particular paragraphs 1 and 4 contain no results – the former summarises the findings, and the latter discusses possible interpretation. There are other sentences in other paragraphs, I suggest separating these and moving to the discussion, to align with STROBE guidance and ensure that the results are easily navigable.

8. “Figure 2 presents the distribution of referral source across the Index of Multiple Deprivation (IMD) in deciles.” This should be figure 1.

9. Figure 2: I suggest converting the proportions from a 0-1 scale to a 0-100% scale, as this will be more familiar to readers. It should not affect any of the findings but will make it easier to read, and aligns with the text itself which uses percentages. I suggest also dropping the t-statistic if possible because readers cannot interpret this.

10. Logistic regression results are presented in terms of AME. Readers may be less familiar with this compared to odds-ratios and I recommend adding more details explaining the meaning of the effect estimates. For example, "This gradient persists even after adjusting for sociodemographic, attendance reasons, clinical and temporal covariates, with the most deprived areas (IMD 1) showing a -0.046 probability difference (p<0.001)." I suggest rephrasing to "4.6 percentage-point lower probability of GP referral, compared to those in IMD 10 postcodes", or similar. This reminds readers of the outcome, reference group, and gives slightly more explanation of the statistic itself.

11. "Referral Source, Deprivation, and ED Outcomes" the first sentence ends with an empty square parenthesis.

12. The interaction analysis of referral source and deprivation was not described in the statistical analysis – I recommend adding so that readers are fully prepared and understand all analyses prior to reading the results.

13. "Using similar regression models but now including interaction terms between IMD deciles and referral sources," As far as I can see, no interaction results are actually presented, it seems to be associations between referral pathway and ED outcomes, stratified by IMD, which is not the same.

14. The statistical analysis reported use of GLM but the results did not mention these, and it is not clear where they were used. The figures are very helpful but some readers may find it easier to read model results from tables, as this tends to be a common way of presenting them, and I suggest the authors consider adding these for the main results.

VERSION 1 - AUTHOR RESPONSE

Reviewer: 1

Comment 1: *Thank you for sharing this manuscript with me. This manuscript addresses an important question using rich data. The manuscript needs improve clarity in some sections and more descriptive tables or figures are needed to help readers understand the whole story. My main comment is you need to explain why you think referral pathways affect downstream outcomes. From economic perspective, perhaps explain from supply and demand of healthcare. From medical perspective, doctors treat patients given the severity of diseases rather than referral routes. Some referral routes may imply less severe diseases so doctors may let them wait longer for the treatment. All in all, you need a theory or framework to support your research questions.*

Answer 1: We thank the reviewer for this thoughtful and constructive feedback. We agree that the manuscript required a clearer theoretical foundation to explain how and why referral pathways may

influence downstream outcomes. In response, we have substantially revised the Introduction (paragraphs 3–4) to introduce a conceptual framework drawing on health services research and access-to-care theory.

Comment 2: *Title: Perhaps think of a title that catches eyes? The current title has two key elements, socioeconomic inequalities and referral pathways, which make me wonder which is the key information that you want to convey?*
Answer 2: We have revised the title to make the main focus clearer and more engaging.

Comment 3 *Introduction: You have mentioned NHS in the first three paragraphs. Given the journal opens to the international readers, perhaps use one or two sentences to explain NHS, just as how you have explained US healthcare system. In the 4th paragraph, you combine aims and contributions together. From logic flow perspective, aims should be in the first place so that it connects well with previous paragraph. Also for the 3rd paragraph, you may consider delete 'however' (it is better to avoid using 'despite' or 'however' as the first word of the first sentence. This is just suggestion; you can stick with your writing style). Still in the 4th paragraph, you address two questions. It would be good mention briefly the framework or the theories that you rely on to address those questions. For example, why different referral sources affect downstream outcomes? If a patient has heart attack and is admitted to ED. Doctors will treat them with priority regardless the referral route they use. Also, some referral routes may be suitable for less emergent diseases. That is, referral sources may be affected by types of diseases, and the downstream outcomes just reflect the differences in diseases not the types of referral routes. Just justify. The last paragraph seems connects well with two questions in the 4th paragraph. Pls consider improving logic flow and coherence.*

Answer 3: We thank the reviewer for this detailed and insightful comment. We have carefully revised the Introduction to improve logical flow, coherence, and clarity. Specifically, we:

1. Added a concise explanation of the National Health Service (NHS) for international readers, clarifying that it is a publicly funded healthcare system providing universal access, with GPs serving as gatekeepers to specialist and emergency care.
2. Reorganised the fourth paragraph to present the study aims first, followed by a clearer outline of the study's contributions, ensuring a smoother connection with the preceding context.
3. Removed "However" from the opening of the third paragraph for stylistic improvement.
4. Introduced a brief theoretical framework drawing on health services research and access-to-care theory to justify why referral pathways may influence downstream outcomes. We explain that referral routes capture differences in both demand-side factors (patient health-seeking behaviour, access barriers) and supply-side factors (triage, coordination, and continuity of care). Clinically, we clarify that while doctors prioritise patients by severity, referral source affects the timing, preparedness, and diagnostic clarity at presentation, which in turn shapes ED process outcomes.

These revisions have substantially improved the logical structure and strengthened the theoretical rationale supporting our research questions.

Comment 4 *Methods: Perhaps in Appendix, you may explain how representative of your sample is. As this is from one hospital, where does it locate (in more or less deprived areas)? Also, the data is from 2019 to 2024, may need a few words to explain whether the pandemic affects your analysis.*
Answer 4: Thanks for pointing this out. We included in the appendix a new figure showing the distribution of the catchment area and IMD (Figure A.6 and A.7) as well as a summary statistic table

(Table A.1). Regarding the time-periods, our models include month-year fixed effects to account for seasonality and changes during and post-pandemic periods.

Comment 5 *In the 2nd paragraph in the Method section, pls consider improving clarity. You use referral sources first as outcomes to examine whether they vary by IMD and then as an explanatory variable to examine whether downstream outcomes vary by referral sources and by IMD. Here, do you mean ten IMD dummies interacted with six referral dummies? All in one equation? It would be easier to explain in equation. The last sentence in this paragraph is redundant.*

Answer 5: That's correct. Our second regression model includes IMD and referral sources as categorical variables. We have now improved clarity by including the corresponding equations.

Comment 6 *For outcomes, as there are international readers, please write a few words that 4 hours is a national target in UK. Also clearly indicate which outcomes are continuous and which are binary, as you mention to use logic and GLM in the later paragraphs.*
Answer 6: Thanks for pointing this out. We have amended the text clarifying the outcome regarding the 4-hour national target.

Comment 7 *Results: I am confused with 1st paragraph, there is no table or figure to show such disparities before reaching ED. If there are main findings, it comes so directly before readers can digest what your data look like. Or your explanation is based on Figure 1? Figure 1 itself is a bit confusing. I don't find arrival mode (lower panel). Is there a typo that Figure 2 should be Figure 1?*
Answer 7: Thank you for your comment. You are correct-there was a typo in that sentence. The explanations in that paragraph were based on Figure 1; and the caption was an old one. We have now amended the text accordingly and also expanded the description of the sample at the start of the Results section to provide readers with a clearer overview before presenting the main findings.

Comment 8 *Also, I am wondering whether to present another figure from alternative angle, the distribution of IMD for each referral source? Similarly, for Figure 2 showing the attendance reasons, is it possible to show within each IMD, the distribution of different reasons. This can help readers to understand different attendance reasons for different IMD. Additionally, the crosstabulation between attendance reasons and referral routes. Figure 2 itself needs more clarity such as what x-axis is?*
Answer 8: Thanks for pointing this out. We have added in the appendix the requested figures (composition of IMD within each referral source, Figure A.8; and composition of IMD within each attendance reason, Figure A.9). As suggested by reviewer 2 too, we have now improved Figure 2's readability by changing the proportion to percentages and adding x-axis title.

Comment 9 *Before showing all those figures, it would be good to just show number of ED visits in each IMD using ranked bar chart. Some studies using data from a single hospital shows much higher visits from IMD10, so worth checking this first.*
Answer 9: Following this suggestion, we have started the result section with a description of the sample. We have also included a summary stats table (Table A.1) in the appendix, as well as additional figures showing the distribution of IMD (A.6, A.7, A.8, and A.9).

Comment 10 *On page 7, the second paragraph, I get lost again as you don't mention which figure I need to read to help me understand your description.*
Answer 10: We agree with the reviewer that the paragraph was confusing, so we decided to remove it.

Comment 11 *T*he 3rd paragraph should be in method section.
Answer 11: We agree with the reviewer and deleted that paragraph from the results section.

Comment 12 *I am a bit confused with the model you use. Am I right that you include IMD dummies with each referral source as an outcome, and IMD10 is the reference group? If so, can you improve clarity?*
Answer 12: That is correct. We have revised the Methods section to improve clarity, specifically in the model description and statistical analysis subsection. We confirm that the Index of Multiple Deprivation (IMD) is treated as a categorical variable, using the least deprived quintile as the reference category. The text now explicitly states how all variables were utilized in the analyses.

Comment 13 Figure 3: pls provide note below the figure (add '0' on x-scale) to help explain the figure. Pls use different legend shapes, considering white and black printing. Also explain why only examine three referral sources rather than six. Explain how you calculate probability (difference between each IMD with IMD10?) Have you detailed the analysis for each attendance reason (the first three common reasons)? Or adding interaction term between IMD and most three common attendance reasons?
Answer 13: Thank you for this comment. As suggested, we have revised the figure to improve clarity. Regarding the referral source outcome, we presented results for the three main referral sources for simplicity and because these (GP/111 service, and Ambulance) have important implications for patient flow and triage in the ED, as well as overall hospital performance. We have now included tables with all referral source outcomes in the appendix.

Comment 14 A)*Figure 4: The title should be hospital admission? I am wondering can you provide a table showing three models: i) IMD as key variable; ii) IMD and referral sources as key variables; and iii) IMD+referral sources+interaction terms.*
Answer 14 A: As suggested, we corrected the title in Figure 4 and reported the full tables in the Supplemental Material. Regarding the interaction model, we note that the inclusion of the IMD deciles (10 categories) and referral sources (6 categories) produces 60 interaction terms, in addition to the main effects and the full set of covariates. Presenting all coefficients for three different models in the manuscript would therefore result in an extremely large and dense table that would be difficult to read and interpret. For clarity, the main text focuses on the marginal effects and key contrasts derived from these models, while the detailed coefficient tables are provided in the Supplemental Material (Tables A.3-A6).

B) *On page 8, Figure 4 should be Figure 5, and the same typo in the following paragraphs.*
Answer B: Thanks for spotting this typo. We have now amended the text and figures.

C) *For Figure 5, it is total time, so should the title and y-axis be different?*
Answer C: We agree with reviewer and changed the title of Figure 5 for total time in ED in minutes. AME represent the average total time in ED for each IMD decile and referral source.

D) *For Figure 4-7: I am wondering whether there is alternative way to show the difference across IMD. Is it possible to use AME as a benchmark, and show difference between AME from each IMD with overall AME?*

Answer D: We appreciate this helpful suggestion. Following the reviewer's advice, we have revised Figures 4–7 to include a horizontal dashed line representing the overall Average Marginal Effect (AME). This line serves as a visual benchmark, allowing for clearer comparison of the AMEs across IMD deciles relative to the overall effect.

Reviewer 2

Comment 1: *The study design, analysis plan and interpretation are all reasonable and I do not see major risks of bias. However, the organization of the paper is somewhat muddled, and this undermines the clarity of the paper. Additionally, the statistical analysis is described very briefly and this makes it challenging to follow the results section. Many of the comments below overlap with the STROBE checklist, which I recommend the authors cross-reference, including the elaboration document which has helpful examples.*

Answer 1: We sincerely thank the statistical reviewer for the positive evaluation of the study design and analytical approach, and for the constructive feedback regarding structure and reporting clarity. We have now reorganised several sections of the manuscript to improve logical flow and readability, especially within the *Introduction* and *Methods* sections. The *Statistical Analysis* section has been substantially expanded to provide a clearer description of the analytical framework, model specifications, covariates, and the interaction analysis between deprivation and referral source. We have also added further detail on the GLM model selection process and distributional assumptions. In addition, we have carefully cross-checked the manuscript against the STROBE checklist and addressed relevant reporting recommendations to enhance transparency and completeness. These revisions improve both the methodological clarity and overall coherence of the manuscript.

Comment 2: *The abstract is for the most part clear, and contains all essential information for readers to get an understanding of the study. However, regarding the statistical reporting I recommend aligning with STROBE guidance and including an uncertainty measure for each comparison (e.g. p-value or 95% CI).*

Answer 2: We have reported the 95% confidence intervals (CIs) for each point estimate mentioned in the abstract.

Comment 3: *"A study using national data found a 50% higher ED attendance rate in the most deprived areas compared to the least deprived" is it possible to also report the absolute percentages for the two groups*?

Answer 3: Thank you for this helpful suggestion. In the referenced study, the distance-adjusted attendance rate increased from approximately 0.45 attendances per person per year in the least deprived areas to 0.68 attendances per person per year in the most deprived areas. This corresponds to a ~50% higher rate in the most deprived groups. We have revised the sentence accordingly to include these absolute values.

Comment 4 *I suggest applying headings to make this easier to follow, such study setting, participants,*

data source/linkage, outcomes, variables, statistical analyses. There is not currently a publication checklist applied and I suggest STROBE.

Answer 4: We thank the reviewer for this suggestion. We agree and have now added headings to the Methods section (Study setting, Participants, Data sources/linkage, Outcomes, Variables, Statistical analyses) to improve clarity and navigability. We have also ensured alignment with the STROBE checklist throughout.

Comment 5 *Participants: How were multiple visits by the same participants handled? Was any deduplication applied such as restricting to the first visit, or were multiple visits permitted? I imagine it is the latter as the unit of analysis is always described as "visits" but it would be good to directly confirm this in the methods.*

Answer 5: We thank the reviewer for this helpful clarification request. Multiple attendances by the same patient were included in the analysis, as the unit of analysis is the *ED visit* rather than the individual patient. Each visit was treated as a distinct observation, consistent with our focus on referral pathways and visit-level outcomes. To account for potential non-independence from repeated visits by the same individuals, we used robust standard errors clustered at the patient level in all regression models. This clarification has now been explicitly added to the *Methods* section.

Comment 6 *The study engages with an interesting question regarding how deprivation and referral pathway interact in ED attendances. This involved testing whether referral pathway varied by deprivation, and evaluating the association of both with various ED-related outcomes. Something which would be very useful is if it was possible for the authors to show the assumed relationship between these variables and the other covariates, i.e. using a DAG, to allow the potential causal relationships to be visualized. This would also help clarify the rationale for the various covariates which are described in paragraph 4 – which are assumed to be confounders, and which are competing exposures adjusted for to reduce uncertainty?*

Answer 6: We thank the reviewer for this very valuable suggestion. We agree that a graphical representation of the assumed relationships would enhance conceptual clarity and make our modelling rationale more transparent. We have therefore added a Directed Acyclic Graph (DAG) (now included as Supplementary Figure A.2) to illustrate the hypothesised relationships between socioeconomic deprivation, referral pathway, ED outcomes, and covariates.

The DAG clarifies that deprivation is conceptualised as an upstream determinant influencing both referral pathway and health status. The referral pathway, in turn, affects downstream ED outcomes (admission, length of stay, breaches, and reattendance). Covariates such as age, gender, and clinical acuity (NEWS) are treated as confounders influencing both referral source and outcomes. Contextual factors (time of attendance, shift type, ED workload) are modelled as competing exposures to reduce residual variance rather than causal confounders. We also revised the Methods section to reference the DAG and clarify the rationale for covariate selection.

Comment 7 *Statistical analysis: this section lacks details which impact the transparency and clarity of the analysis, and it would be helpful to explain (a) how each model aligns with the aims or how the result is intended to be interpreted, (b) the full specification of each model, i.e. the outcome and covariates and (c) more information about the GLM – which family specifications were considered, and how was non-normality determined and for which outcomes.*

Answer 7: Thanks for pointing this out. Following your suggestions and the ones of Reviewer 1, we have revised the entire Methods section to clarify the objectives of each analysis and how the results are intended to be interpreted. We have now provided the full specification of each model, including outcomes and covariates, and added more details about the GLM, including the family specifications considered (GLM with a log link and Gamma family to model total minutes in the ED) and how non-normality was assessed for each outcome.

Comment 8 *There are certain elements which seem more suitable to the discussion, as the focus is on interpreting findings rather than presenting them. In particular paragraphs 1 and 4 contain no results – the former summarises the findings, and the latter discusses possible interpretation. There are other sentences in other paragraphs, I suggest separating these and moving to the discussion, to align with STROBE guidance and ensure that the results are easily navigable.*

Answer 8: We thank the reviewer for this suggestion. We agree and have now revised the entire Results section, limiting it to the presentation of findings and moving any interpretation or discussion of implications to the Discussion section, in line with STROBE guidance.

Comment 9 "Figure 2 presents the distribution of referral source across the Index of Multiple Deprivation (IMD) in deciles." This should be figure 1.
Answer 9: Thanks for spotting this typo. We have amended the text and caption for that figure.

Comment 10 Figure 2: I suggest converting the proportions from a 0-1 scale to a 0-100% scale, as this will be more familiar to readers. It should not affect any of the findings but will make it easier to read and aligns with the text itself which uses percentages. I suggest also dropping the t-statistic if possible because readers cannot interpret this.
Answer 10: As suggested, we changed the proportions to percentages. We also added x-axis title and horizontal line showing the range of percentages.

Comment 11 Logistic regression results are presented in terms of AME. Readers may be less familiar with this compared to odds-ratios and I recommend adding more details explaining the meaning of the effect estimates. For example, "This gradient persists even after adjusting for sociodemographic, attendance reasons, clinical and temporal covariates, with the most deprived areas (IMD 1) showing a -0.046 probability difference ($p<0.001$)." I suggest rephrasing to "4.6 percentage-point lower probability of GP referral, compared to those in IMD 10 postcodes", or similar. This reminds readers of the outcome, reference group, and gives slightly more explanation of the statistic itself.
Answer 11: We thank the reviewer for this suggestion. We agree and have added more detail in the Methods section explaining the rationale for using AMEs. In addition, we have revised the Results to report point estimates as suggested, providing percentage-point differences alongside the reference group to improve clarity and interpretability.

Comment 12 "Referral Source, Deprivation, and ED Outcomes" the first sentence ends with an empty square parenthesis.
Answer 12: Thanks for spotting this typo. We have now removed the empty square parentheses.

Comment 13 The interaction analysis of referral source and deprivation was not described in the statistical analysis – I recommend adding so that readers are fully prepared and understand all analyses prior to reading the results.
Answer 13: We have included now a description of the interaction analysis.

Comment 14 "Using similar regression models but now including interaction terms between IMD

deciles and referral sources," As far as I can see, no interaction results are actually presented, it seems to be associations between referral pathway and ED outcomes, stratified by IMD, which is not the same.

Answer 14: We appreciate the reviewer's careful reading and helpful comment regarding the presentation of interaction effects. We confirm that our regression models do indeed include interaction terms between IMD deciles and referral source (specified in Stata as b10.imd10##i.referral). To ensure correct interpretation in a nonlinear model (logit), we followed established guidance by presenting average marginal effects (AMEs) and predicted probabilities derived from those interaction models; rather than relying solely on raw interaction-term coefficients. Specifically, we followed the approach recommended in the Stata documentation: "margins and marginsplot for the interaction of two categorical predictor variables" (StataCorp,https://www.stata.com/links/stata-basics/margins-marginsplot-interaction-two-categorical-predictor/). This ensures that the effect of one variable (IMD decile) is correctly shown conditional on the categories of the interacting variable (referral source), and vice versa. We have also revised the manuscript text and figure captions to make this clearer for readers.

Comment 15 The statistical analysis reported use of GLM but the results did not mention these, and it is not clear where they were used. The figures are very helpful but some readers may find it easier to read model results from tables, as this tends to be a common way of presenting them, and I suggest the authors consider adding these for the main results.

Answer 15: We used the GLM for the length of stay in the ED (in minutes) as this outcome has a skewed distribution with a long tail.

We have clarified this in the method section. We also report full results tables in the appendix section.

VERSION 2 - REVIEW

**Reviewer**	**1**
**Name**	**Gao , Ni**
**Affiliation**	**University of York**
**Date**	**20-Nov-2025**
**COI**	

Many thanks for the great effort! The manuscript is now written in a very clear way. There are two tiny comments: i)title, ED should be emergence department; ii) When you introduce NHS, you written the same sentence in the first and third paragraph, pls consider removing one of them.

**Reviewer**	**2**

**Name**	**Dack, Kyle**
**Affiliation**	**University of Bristol, MRC Integrative Epidemiology Unit**
**Date**	**13-Nov-2025**
**COI**	

Thank you to the authors for the improvements made to the manuscript, and their responses, which together answer all queries and suggestions I had in the first review.

I have read the manuscript again and the methods and statistics are all aligned with STROBE, and the paper looks suitable for publication.

My only minor comment which perhaps could be addressed during proofing is to consider add more paragraphs to improve readability, make it easier for readers to locate information and understand when new ideas are being introduced.

For example: "Methods: Data and participants" – new paragraphs could be added where the topic changes considerably on the lines "The sample covers the period from January 2019 to December 2023", and "The dataset includes all Emergency Department (ED) attendances by patients aged 16 and over."

VERSION 2 - AUTHOR RESPONSE

Response to Reviewer 2

Comment:
The manuscript is improved and methods and statistics align with STROBE. A minor suggestion is to add more paragraphs to improve readability, particularly in the section "Methods: Data and participants." New paragraphs could be added where the topic changes, for example at the sentences beginning "The sample covers the period from January 2019 to December 2023" and "The dataset includes all Emergency Department (ED) attendances by patients aged 16 and over."

Response:
Thank you very much for this thoughtful comment and for your positive assessment of the revised manuscript. We agree that additional paragraph breaks would enhance readability. We have now added paragraph breaks along the text

---

Response to Reviewer 1

Comment i:
In the title, ED should be written out as Emergency Department.

Response:
Thank you for spotting this. We have revised the title so that the abbreviation "ED" is

now written in full as **“Emergency Department.”** This ensures clarity for all readers, including those less familiar with clinical abbreviations.

Comment ii:
The sentence introducing the NHS appears both in the first and third paragraph; please consider removing one.

Response:
We appreciate this helpful observation. We have removed the duplicated introductory sentence about the NHS from the third paragraph, retaining only the earlier version where it fits more naturally

---
